# Effects of gastric bypass bariatric surgery on gut microbiota in patients with morbid obesity

**DOI:** 10.1080/19490976.2024.2427312

**Published:** 2024-11-17

**Authors:** Urja Amin, Dora Huang, Arun Dhir, Anya E Shindler, Ashley E Franks, Colleen J Thomas

**Affiliations:** a Department of Microbiology, Anatomy, Physiology and Pharmacology, La Trobe University, Bundoora, Victoria, Australia; b Centre for Cardiovascular Biology and Disease Research, La Trobe University, Bundoora, Victoria, Australia; c Department of Surgery, Austin Health, Heidelberg, Victoria, Australia; d Body Genesis Institute, Bundoora, Victoria, Australia; e Pre-Clinical Critical Care Unit, Florey Institute of Neuroscience and Mental Health, University of Melbourne, Parkville, Victoria, Australia

**Keywords:** Obesity, gastrointestinal microbiota, bariatric surgery, Roux-en-Y gastric bypass, one anastomosis gastric bypass

## Abstract

The Western diet is associated with gastrointestinal dysbiosis, an active contributor to the pathophysiology of obesity and its comorbidities. Gastrointestinal dysbiosis is strongly linked to increased adiposity, low-grade inflammation, dyslipidaemia, and insulin resistance in individuals with morbid obesity. Bariatric bypass surgery remains the most effective treatment for achieving significant weight loss and alleviating obesity-related comorbidities. A growing body of evidence indicates that traditional Roux-en-Y Gastric Bypass (RYGB) improves the disrupted gut microbiota linked with obesity, potentially contributing to sustained weight loss and reduction of comorbidities. One Anastomosis Gastric Bypass (OAGB), a relatively new and technically simpler bariatric procedure, has shown both safety and efficacy in promoting weight loss and improving comorbidities. Few studies have investigated the impact of OAGB on gut microbiota. This review provides insights into the pathogenesis of obesity, current treatment strategies and our current understanding of the gut microbiota in health and disease, including modulating the gut microbiota as a promising and novel way to alleviate the burden of obesity and cardiometabolic conditions. By exploring the impact of gastric bypass surgery on gut microbiota-host interactions, we aim to shed light on this evolving field of research and uncover potential therapeutic targets for elevating outcomes in bariatric surgery.

## GRAPHICAL ABSTRACT

**Figure:**
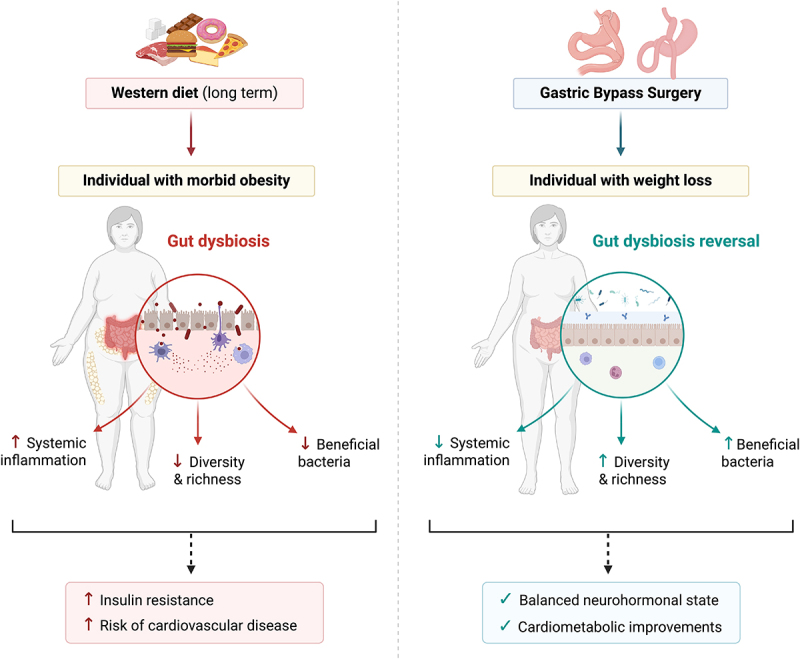

## Introduction

The global obesity epidemic stems from sedentary lifestyles and unhealthy eating patterns. Mounting evidence suggests that disruptions in the normal composition and function of gastrointestinal bacteria are closely associated with obesity and associated metabolic disorders, including insulin resistance and low-grade inflammation.^1,2^ Consequently, restoring a balanced microbiota is increasingly recognized as a promising strategy for preventing and managing obesity.^2^ Bariatric surgery has emerged as a highly effective treatment for Class III obesity (morbid obesity), leading to significant weight loss and improvement in obesity-related health issues. Of the two surgical options, Roux-en-Y Gastric Bypass (RYGB) and One-Anastomosis Gastric Bypass (OAGB), the former is the most extensively studied and shown to restore gut microbial balance and enhance metabolic outcomes.^3^ The newer and technically simpler OAGB procedure has gained popularity due to its favorable outcomes and reduced complication rates. However, its impact on gut microbiota has yet to be thoroughly investigated. This review presents the latest evidence regarding the intricate interplay between obesity and gut microbiota, current understanding of post-RYGB gut microbiota restoration and knowledge gaps regarding OAGB and the gut microbiome. By exploring this evolving field, we hope to gain and share insights into the mechanisms through which gastric bypass surgery interacts with the intricate composition and functionality of gut microbiota, shedding light on potential therapeutic targets for enhancing metabolic outcomes.

## The state of obesity and cardiometabolic comorbidities

Nutritional excess from prolonged consumption of a poor diet, such as a western diet (rich in saturated fats, refined carbohydrates, salt and low fiber intake), is a leading contributor to visceral fat accumulation.^4^ This accumulation plays a key role in the development of metabolic syndrome (MetS) which is a cluster of metabolic disorders that significantly increase the risk of stroke and cardiovascular disease (CVD). Risk factors associated with MetS include obesity (body mass index (BMI) >30 kg/m^2^), hyperglycemia, hypertension and atherogenic dyslipidaemia.^5^ Adiposity, chronic low-grade inflammation, insulin resistance, hyperlipidemia, and neurohormonal activation all play significant roles in the progression of obesity and its association with Type 2 Diabetes Mellitus (T2DM), MetS, and CVD. These mechanisms have been extensively discussed in other reviews (eg,^6–11^) but are highlighted briefly below.

## Increased adiposity and systemic inflammation

Adipose tissue plays a central role in energy homeostasis and metabolic dysfunction. Comprised mainly of adipocytes, it stores energy as lipid droplets within a fibrous network and acts as an endocrine organ to secrete hormones and cytokines.^7^ Adipocytes convert free fatty acids (FFA) into triglycerides (lipogenesis) after eating. During high metabolic demand, lipolysis releases FFAs and glycerol to generate energy.^12^ These processes, predominantly regulated by hormonal pathways, reflect the central role of adipose tissue in maintaining whole-body energy homeostasis.

**Figure 1.**
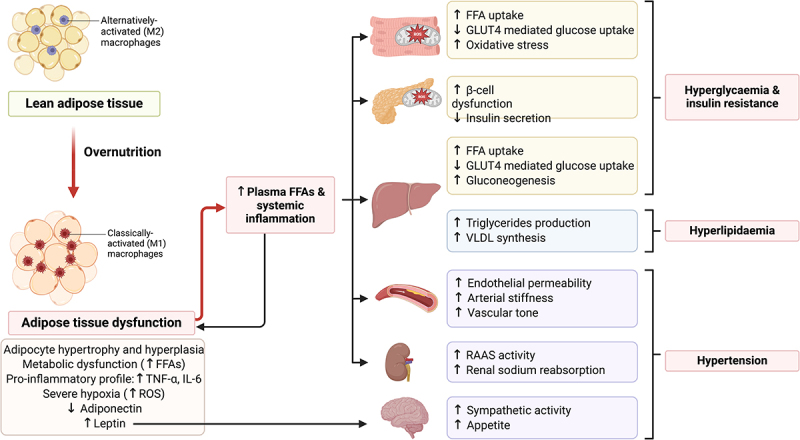
Mechanisms underlying obesity-induced metabolic dysfunction. Obesity triggers a shift in macrophage activation from the protective M2 to the maladaptive M1 state. Dysfunctional adipocytes release elevated plasma free fatty acids (FFA) levels and inflammatory mediators, such as tumor necrosis factor-alpha (TNF-α) and interleukin-6 (IL-6) that induce systemic inflammation. Impaired glucose uptake via glucose transporter type-4 (GLUT4) induces oxidative stress in skeletal muscle, leading to reduced insulin sensitivity. Chronically increased plasma FFA stimulates gluconeogenesis, induces hepatic insulin resistance, and impairs insulin secretion. Under constant inflammatory environment, pancreatic β-cells, undergo mitochondrial stress, impairing insulin secretion. This promotes a hyperglycaemia, and insulin resistance state, eventually progressing to type 2 diabetes. FFAs uptake in the liver stimulates triglyceride production and very-low-density lipoprotein (VLDL) synthesis, causing hyperlipidemia. Additionally, adipocyte dysfunction and systemic inflammation contribute to vascular endothelial dysfunction and renin-angiotensin-aldosterone system (RAAS) activation, which play a role in the development of chronic hypertension. Elevated leptin levels further enhance sympathetic activity and appetite, potentially worsening hypertension. Created with BioRender.com.

Adipose tissue dysfunction is one of the initial mechanisms triggering inflammation in an individual with poor diet. Excessive accumulation of FFAs within adipocytes, consequently promotes the expansion of adipose tissue through a combination of adipocyte enlargement (hypertrophy) and increased adipocyte number (hyperplasia).^6,13^ Greater FFA content within adipocytes exacerbates oxidative processes, including lipid peroxidation, resulting in hypoxia and cellular oxidative stress characterized by elevated levels of reactive oxygen species (ROS).^6,13^ As a consequence, many immune cells, including macrophages, are recruited to the adipose tissue. In individuals with obesity, the interplay between nutritional excess and an inflammatory environment drives macrophage activation from a protective alternative (M2) state to a maladaptive classical (M1) state.^14^ The infiltration of classically (M1)-biased adipose tissue macrophages initiates a local inflammatory environment characterized by an increased release of pro-inflammatory cytokines (e.g., tumor necrosis factor-alpha (TNF-α), interleukin-6 (IL-6)), and leptin levels, and reduced secretion of anti-inflammatory adipokines, including adiponectin and interleukin-10.^6,14^ Ultimately, under inflammatory and stressful conditions, adipocytes undergo apoptosis.^10,14^ The accumulation of dysfunctional adipocytes, combined with the release of pro-inflammatory cytokines sets the stage for systemic low-grade inflammation, a hallmark of obesity-associated metabolic complications (Figure 1).

## Development of insulin resistance and dyslipidemia

Obesity is a risk factor for poor glycemic control and the development of T2DM. Adipose tissue in obese individuals is under chronic metabolic stress and releases pro-inflammatory cytokines and FFAs due to elevated lipolysis, while release of adiponectin, an anti-inflammatory insulin-sensitizing adipokine, is reduced.^15^ Chronically, elevated systemic FFA influx, deposited as ectopic fat, causes lipotoxicity in peripheral tissues including skeletal muscle, adipocytes and liver, leading to downstream inhibition of insulin signaling required for activation of insulin-dependent glucose transport. This results in reduced translocation of the glucose transporter type-4 (GLUT4) to the plasma membrane and glucose uptake.^9^ Inhibition of insulin signaling and reduced plasma adiponectin levels, elevates hepatic gluconeogenesis, resulting in increased hepatic glucose output. Insulin unable to supress clearance of FFAs and lipolysis in adipocytes, creates a constant negative feedback loop.^8^ This progressive loss of insulin sensitivity leads to hyperglycemia, promoting demand for pancreatic β-cells to expand, produce and secrete more insulin as a compensatory mechanism.^9^ The hyperinsulinemia response by β-cells initially restores plasma glucose levels in the prediabetic stage. However, chronic exposure to FFAs and proinflammatory cytokines induces mitochondrial oxidative stress, releasing ROS in pancreatic β-cells.^16^ As a result, β-cell mass is reduced leading to β-cell dysfunction and/or apoptotic cell death. Insulin secretion decreases and consequently raises blood glucose levels.^16,17^ A persistent hyperglycemic and hyperinsulinemia state, induces systemic insulin resistance in patients with obesity, eventually progressing to T2DM.

Adipose dysfunction, inflammation and insulin resistance establish a vicious cycle which perpetuates metabolic dyslipidemia. Dyslipidemia is characterized by elevated triglyceride levels accompanied by reduced high-density lipoprotein (HDL) concentrations.^5^ Briefly, insulin-resistant adipocytes secrete increased levels of FFAs into the systemic circulation, leading to triglyceride accumulation in the liver. In response, increased very-low-density lipoprotein (VLDL) assembly and secretion elevates hepatic triglyceride export, ultimately resulting in hyper-triglyceridemia.^18^ Reduced lipoprotein lipase activity in dysfunctional adipocytes, skeletal muscle and liver hinders the clearance of FFAs and VLDL particles.^18^ Triglycerides are transferred from VLDL to both HDL and low-density lipoprotein (LDL), resulting in triglyceride-enriched HDL and LDL particles. Triglyceride-enriched HDL is rapidly cleared by the kidneys, reducing its capacity to remove cholesterol from blood vessels.^10^ This hyper-triglyceridemic state with reduced systemic HDL constitutes the hallmark of the atherogenic dyslipidemia in patients with obesity.

## Hypertension

Hypertension (i.e., systolic blood pressure >140 mmHg and diastolic blood pressure >90 mmHg) is a common comorbidity of obesity.^5^ The development of obesity-induced hypertension involves a complex interplay of neurohumoral and renal mechanisms yet to be fully understood. Leptin, a hormone secreted by adipocytes, appears to play a role in energy regulation by stimulation of sympathetic nervous system (SNS) to decrease appetite, and increase energy expenditure, and thus increasing blood pressure.^19^ Individuals with obesity have high circulating leptin levels.^20,21^ Selective leptin-resistance has been suggested to occur in patients with obesity, as leptin’s metabolic effects (promoting satiety and weight loss) become ineffective whereas leptin-mediated sympathetic activation is preserved, possibly contributing to the pathogenesis of obesity-related hypertension.^21–23^

Another mechanism for hypertension in patients with obesity involves the prolonged activation of the renin-angiotensin-aldosterone system (RAAS).^11,24,25^ In obesity, enlarged dysfunctional adipocytes secrete higher levels of angiotensinogen and angiotensin II, leading to the overactivation of both the SNS and RAAS.^24,25^ The excessive activation as well as physical compression of the kidneys due to visceral adiposity, results in increased renal tubular sodium reabsorption, impaired renal pressure natriuresis and volume expansion, causing hypertension.^11,24^ Additionally, systemic inflammation, elevated proinflammatory cytokines, and ROS production further contribute to vascular endothelial dysfunction.^26^ Consequences of vascular endothelial dysfunction includes enhanced vasoconstriction, increased vascular endothelial permeability, oxidation of LDL, and sub-endothelial modifications, driving vascular endothelium toward pro-thrombotic and pro-atherogenic states.^23,27^ Artery narrowing due to plaque accumulation acts as a compensatory mechanism, elevating blood pressure. Chronic obesity-induced hypertension damages blood vessel walls, increasing the risk of atherosclerosis, reduced blood supply to tissues and organs and heightened susceptibility to CVD or stroke.

## Treatment options for patients with morbid obesity

Adopting a healthy diet and regular exercise remains the cornerstone advice for patients experiencing obesity and its related comorbidities. For effective weight loss, a recommended calorie-restricted diet and moderate intensity physical activity of at least 45–60 minutes per day may need to be complemented by available surgical and non-surgical modalities.^28^ Patients who fail to lose weight or regain weight despite calorie restriction or increased exercise may benefit from pharmacotherapy, which includes appetite suppressor drugs (anorectics). The Therapeutic Goods Administration (TGA) have approved phentermine, liraglutide, semaglutide and bupropion-naltrexone for suppressing appetite through central nervous system actions, while orlistat inhibits fat absorption by inhibiting gastric and pancreatic lipases.^29–32^ It should be noted that weight loss via pharmacotherapy is often accompanied by unwanted side effects (e.g., dry mouth, dizziness, nausea, diarrhea, pancreatitis) and can impose financial burden on individuals due to the high cost of medications.^33^

Individuals may resort to over-the-counter weight-loss supplements, to achieve their weight-loss goals. The most common ingredients in these supplements are include Irvingia gabonensis (known for its adipogenesis-inhibiting properties), caffeine, and green tea extract, which are suggested to enhance energy expenditure and promote fat oxidation. A significant limitation of these supplements is their lack of supporting efficacy and safety data.^34^ Individuals with obesity can also benefit from calorie-restricted meal replacement shakes or snack bars.^35,36^ A recent randomized trial of patients with morbid obesity found very-low calorie meal replacement OPTIFAST shakes program for 52 weeks achieved superior clinical weight loss of 18 ± 9% total body weight-loss compared to 12 ± 7% achieved by intragastric balloon placement.^37^

## Bariatric surgery

Despite attempts at lifestyle modifications or medically supervised interventions (e.g., drug/supplement/meal replacement shakes), some patients with obesity are unable to lose weight or maintain their weight loss.^3^ For such individuals, bariatric surgery is considered a viable option to expedite their weight loss.^3^ Bariatric surgery is recommended for individuals diagnosed as morbidly obese (Class III obesity; BMI >40 kg/m^2^) or severely obese (Class II obesity; BMI >35 kg/m^2^) with serious obesity-related complications.^38^

Common and well-established procedures in bariatric surgery include sleeve gastrectomy (SG), adjustable gastric banding, and RYGB. Among these procedures, RYGB has been found to be particularly effective, leading to rapid weight loss, reduced adiposity, and improved glucose metabolism.^39^ First described by Mason and Ito in 1966, the RYGB operation includes creating a small gastric pouch by partitioning the stomach aiming to reduce food intake and promote satiety.^40^ A biliopancreatic limb is created, comprised of the duodenum and proximal jejunum, which stays proximally attached to the remaining stomach. This biliopancreatic limb is then anastomosed to the distal section of the jejunum, resulting in a jejunojejunostomy (jejunojejunal anastomosis) that allows the flow of bile and pancreatic enzymes required for digestion to continue. The Roux limb is typically 75 to 150 cm long from the place of jejunal division and attached to the small gastric pouch, resulting in a gastrojejunostomy (gastrojejunal anastomosis).^41^ Food can bypass most of the stomach and the upper portion of the small intestine. This re-routing of the gastrointestinal tract modifies nutritional absorption and processing, resulting in weight loss and metabolic alterations.

### One anastomosis gastric bypass

A relatively new reversible laparoscopic procedure that has become increasingly popular amongst bariatric surgeons is called the one anastomosis gastric bypass (OAGB). In 2001, Rutledge proposed the use of a single anastomosis to simplify the RYGB surgical technique, known as ‘mini’ gastric bypass.^42^ In 2002, Carbajo performed a modification of the MGB to avoid potential reflux, naming it OAGB.^43^ The purpose of OAGB is to reduce operative time, lower the risk of complications, achieve faster patient recovery and reduce morbidity, yet achieve comparable outcomes to the previous gold standard RYGB procedure.^44^ The International Federation for the Surgery of Obesity and Metabolic Disorders has agreed that the standard nomenclature for this procedure should be the mini gastric bypass-one anastomosis gastric bypass (MGB-OAGB).^45^

**Figure 2.**
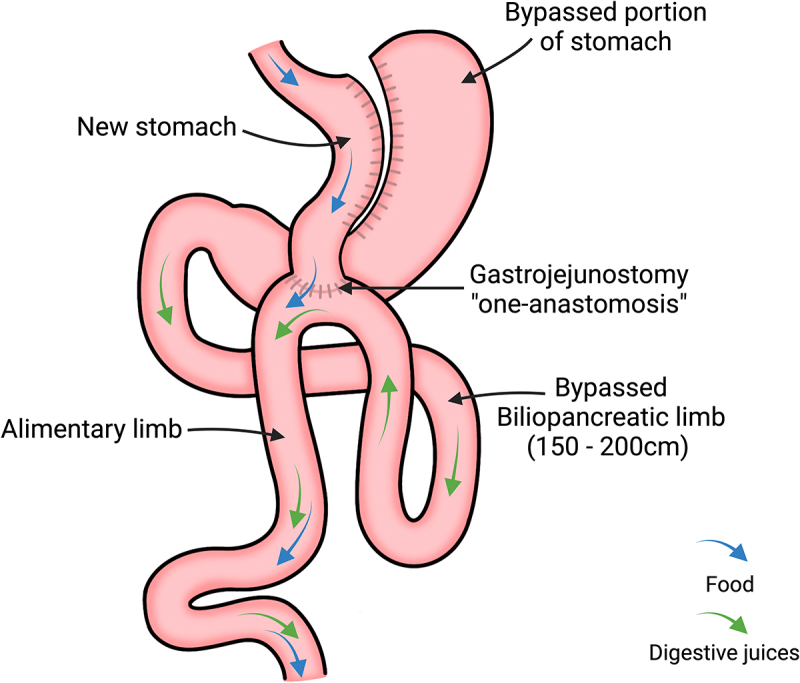
One-anastomosis gastric bypass (OAGB) surgical approach. The stomach is divided to create a smaller stomach pouch and reroutes the small intestine to this pouch. Of benefit to the patient, OAGB only involves one surgical connection between the new stomach and the alimentary limb, a distal portion of the jejunum (gastrojejunal anastomosis or gastrojejunostomy). Most of the stomach and biliopancreatic limb (proximal segment of the small intestine that is connected to the stomach, pancreas, and hepatic system) is bypassed. As a result, when food is consumed, it first enters the “new” stomach, followed by the alimentary limb, and into the colon, bypassing the upper part of the intestinal tract. Created with BioRender.com.

The laparoscopic OAGB procedure results in a physical limitation in the quantity of food that can be ingested, and modifies energy and nutrient absorption, both leading to improved metabolic outcomes. As illustrated in Figure 2, the top of the stomach is stapled to form a thin tube that becomes the new, smaller stomach and is completely separated from the remnant stomach. This newly formed stomach limits the amount of food that can be consumed. Gastrojejunal anastomosis refers to the surgical connection between the stomach pouch and the small intestine. The new stomach pouch is anastomosed to the alimentary limb which is the middle portion of the small intestine (the jejunum), bypassing 150–200 cm of the biliopancreatic limb. Depending on the surgeon’s preference and the patient’s anatomy, the exact technique for creating the gastrojejunal anastomosis may vary. As a result, when food is consumed, it first enters the “new” stomach and followed by the jejunum, “bypassing” the upper part of the intestinal tract. Bypassing the upper portion of the intestinal tract results in a decrease in the absorption of calories and nutrients. The unused portions of the stomach and intestine are still present in the body; however, they no longer contribute to digestion.

Table 1 summarizes the key findings from recent eight distinct studies, collectively highlighting the effectiveness of OAGB in addressing obesity and associated comorbidities. Despite differences in sample sizes, patient demographics, and follow-up durations, a consistent trend emerges: OAGB results in significant weight loss, improved lipid profiles, and glycemic control, underscoring its effectiveness for a diverse range of patients.^46–52^ The procedure often demonstrates outcomes comparable to or surpassing other bariatric procedures like RYGB and SG.^48–53^ OAGB has proven to be an effective alternative for individuals who have experienced limited success or complications with restrictive bariatric surgery.^54–56^ Thus, previous literature has demonstrated that patients with morbid obesity undergoing OAGB experience substantial weight reduction and improvements in obesity-related comorbidities.

**Table 1.** Recent prospective cohort studies on OAGB, weight loss and resolution of comorbidities.

Study	Population	Procedure(s)	Follow-up period	Findings
Kruschitz et al.^46^ (Italy)	37 patients (mean age 42 years, 80% females, weight 120 ± 13 kg, BMI 44 ± 4 kg/m^2^).	OAGB	1 year	Significant weight loss to 78 ± 11 kg. *** Significantly reduced BMI to 28 ± 4 kg/m^2^. *** Significantly reduced SBP (−14 mmHg). *** Significantly improved lipid profile (HDL +7 mg/dl; triglycerides −67 mg/dl; LDL −30 mg/dl). ***
Kessler et al.^47^ (Israel)	50 patients (mean age 46 years, 72.1% women, weight 115 ± 18 kg, BMI 42 ± 5 kg/m^2^).	OAGB	12-20 months	Significant weight loss to 75 ± 13 kg. *** Significantly reduced BMI to 27 ± 4 kg/m^2^. *** Significantly improved glycemic status (HbA1c and glucose). *** Tended to improve lipid profile (reduced triglycerides, total cholesterol, LDL, and increased HDL levels).
Bettini et al.^48^ (Italy)	46 OAGB patients (mean age 47 years, weight 124 kg, BMI 46 kg/m^2^). 88 LSG patients (mean age 48 years, weight 124 kg, BMI 45 kg/m^2^).	OAGB vs SG	18 months	OAGB achieved greater significant weight loss by −33% (mean 83 kg ***) compared to −30% (mean 92 kg ***) with SG.* OAGB achieved greater significant BMI reduction by −34% (mean 30 kg/m^2^ ***) compared to −29% (mean 34 kg/m^2^ ***) with SG.* Plasma lipid levels were significantly lower post-OAGB compared to SG, thus superior improvement in dyslipidaemia.***
Robert et al.^49^ (France)	117 OAGB patients (mean age 44 years, 73% females, weight 121 ± 24 kg, BMI 44 ± 6 kg/m^2^). 117 RYGB patients (mean age 43 years, 78% females, weight 120 ± 19 kg, BMI 44 ± 5 kg/m^2^).	OAGB vs RYGB	2 years	OAGB achieved a significantly greater weight loss by −37% compared to −35% in RYGB patients. *** OAGB achieved a significantly greater BMI reduction of −88 ± 24% compared to −86 ± 23% with RYGB. ** OAGB achieved significantly greater HbA1C reduction by −2 ± 2% compared to −1 ±1% with RYGB. *
Singh et al.^50^ (India)	25 OAGB patients (mean age 46 years, weight 120 ± 22 kg, BMI 47 ± 7 kg/m^2^, 69% hypertensive patients). 24 RYGB patients (mean age 47 years, weight 113 ± 16 kg, BMI 45 ± 5 kg/m^2^).	OAGB vs RYGB	4 years	OAGB achieved greater % EWL by −58% compared to −53% with RYGB. RYGB had a greater BMI fall to 33 kg/m^2^ compared to 34 kg/m^2^ with OAGB. OAGB achieved a T2DM remission rate of 72% compared to 71% with RYGB.
Rheinwalt et al.^51^ (Germany)	324 OAGB patients (mean age 43 years, 75% female, BMI 54 ± 7 kg/m^2^, 69% hypertensive patients). 288 RYGB patients (mean age 41 years, 80% females BMI 45 ± 4 kg/m^2^, 60% hypertensive patients).	OAGB vs RYGB	3 years	OAGB achieved greater significant total weight loss of −36 ± 9 kg compared to −34 ± 9 kg with RYGB. * RYGB achieved higher hypertension remission rate 71% versus 58% in OAGB patients.
Level et al.^52^ (Venezuela)	9 OAGB patients (mean age 38 years, weight 110 ± 16 kg, BMI 43 ± 6 kg/m^2^). 19 RYGB patients (mean age 37 years, weight 109 ± 20 kg, BMI 43 ± 6 kg/m^2^).	OAGB vs RYGB	5 years	OAGB achieved greater significant weight loss of −48% (67 ± 9 kg ***) compared to RYGB −42% (71 ± 7 kg ***). OAGB achieved greater significant BMI reduction of −55% (24 ± 1 kg/m^2^ ***) compared to RYGB −51% (25 ± 3 kg/m^2^ ***). Similar resolution of comorbidities; OAGB: T2DM (11%), hypertension (44%), dyslipidaemia (11%); RYGB: T2DM (11%), hypertension (31%), dyslipidaemia (11%).
Bhandari et al.^53^ (India)	90 OAGB patients (mean age 44 years, 33% female, weight 131 ± 24 kg, BMI 46 ± 7 kg/m^2^). 122 RYGB patients (mean age 44 years, 47% female, weight 115 ± 20 kg, BMI 42 ± 6 kg/m^2^).	OAGB vs RYGB	5 years	OAGB achieved a significantly greater total weight loss by −35 ± 7% compared to −24 ± 7% in RYGB patients. *** OAGB tended to have higher resolution of comorbidities; OAGB: T2DM (79%), hypertension (56%), dyslipidaemia (56%), sleep apnea (95%); RYGB: T2DM (62%), hypertension (43%), dyslipidaemia (54%), sleep apnea (91%).

Data expressed as mean ± standard deviation and rates (%).

Significant changes indicated by: *p ≤ 0.05; **p ≤ 0.01; ***p ≤ 0.001.

Abbreviations: BMI, Body Mass Index. EWL, Excess Weight Loss. HbA1c, Haemoglobin A1c. HDL, High-density Lipoprotein. LDL, Low-density Lipoprotein. OAGB, One Anastomosis Gastric Bypass. RYGB, Roux-en-Y Gastric Bypass. SG, Sleeve Gastrectomy. T2DM, Type 2 Diabetes Mellitus.

### Risk profile of OAGB

Despite the growing popularity of OAGB due to its simplicity and clinical benefits, the procedure carries a comparable risk profile to RYGB, with malnutrition being a common concern due to its malabsorptive component. The recent YOMEGA trial found that 9 out of 129 patients who underwent OAGB experienced nutritional complications, while no such complications were reported in the RYGB group (*n* = 124).^52^ Nutritional deficiencies, particularly anemia, vitamin D deficiency, and low hemoglobin levels, have also been reported in patients undergoing OAGB, alongside gastrointestinal side effects such as flatulence (68%) and diarrhea (26%).^51^ The majority of patients who experience nutritional complications respond well to nutritional supplements, without requiring immediate intervention or conversion procedures.

OAGB has fewer early and late surgical complications (4% to 7.5% of cases) compared to RYGB, attributed to shorter operative time and the simplicity of the surgical technique.^57^ However, complications like leaks (approximately 1.5% of cases) and bile reflux (7.8% to 55.5% cases) still occur.^58,59^ Keleidari et al. found bile reflux incidence rates 12 months after surgery were similar between OAGB and RYGB (7.8% and 3.4% respectively).^60^ Common early post-operative symptoms experienced by patients include abdominal pain, frequent heartburn, nausea, and vomiting. In rare cases, postoperative complications like marginal ulcer, dumping syndrome, small bowel obstruction, internal hernia, and stomal stenosis may arise.^61,62^ Evidence indicates insufficient weight loss is uncommon following OAGB, affecting only 1.7% of patients.^63^ For these patients, surgeons and dietitians work closely together to evaluate the individual case and decide on the best course of action. This may involve modifying their diet or performing revisional surgery to address the issue of inadequate weight loss effectively.^63^

### Post-operative changes

Gastric bypass surgery modifies the anatomy and physiology of the gastrointestinal tract by reducing gastric volume, modifying nutrient absorption dynamics and influencing energy expenditure.^64,65^ Secretions and actions of gut hormones are also altered, including increased release of satiety-promoting gut hormones such as glucagon-like peptide 1 (GLP-1) and peptide YY (PYY).^65^ Post-operative changes from bariatric surgery have also been summarized as the so-called BRAVE effect (Bile flow alteration, Reduction of gastric size, Anatomical gut rearrangement and Altered flow of nutrients, Vagal manipulation, and Enteric hormones modulation).^66^

There is emerging evidence that bariatric surgery also significantly influences gut microbial-host metabolic crosstalk, resulting in significant weight loss, improved lipid profiles, reduced insulin resistance, and inflammation in patients with obesity.^67^ These changes reduce the risk of MetS, suggesting that the gut microbiota plays an influential role in the pathophysiology of obesity and obesity-related comorbidities.^1^

## The human gastrointestinal microbiota: a complex ecosystem

Bacteria, viruses, and fungi are among the trillions of microorganisms that make up the gastrointestinal microbiota, an ecological community in the intestinal tract.^2^ A symbiotic and mutualistic relationship exists between the gut microbiota and the host (human). Microbial colonization begins immediately at the birth of an infant.^68^ Gut microbiota development can be affected by genetics and a range of early life events including the mode of delivery (cesarean section or vaginal), feeding methods (breastfed, or formula, or mixed fed), discontinuation of breast-feeding, and external factors such as maternal medication use, especially antibiotics.^69,70^

The development of the human gut microbiota in childhood has long-term effects on the host that may have an impact on health in adulthood.^70^ Although the gut microbiota is influenced by a variety of factors, including diet, lifestyle (eg. exercise, alcohol, smoking), geographical location, medication, and disease, it becomes more stable and diverse over the years in healthy adults. In a healthy individual, the gut microbiota is dominated primarily by the phyla Firmicutes and Bacteroides, with minor levels of Actinomycetota, Verrucomicrobiota and Pseudomonadota.^71^ In spite of certain enterotypes, human gut microbiomes differ greatly between individuals, and some of these differences are associated with chronic health conditions.^72^ Patients with autoimmune diseases, including rheumatoid arthritis, exhibit a modified gut microbiota composition, which has been suggested to play a role in disease progression.^73,74^ Altered gut microbiota is linked to persistent overactivation of innate and adaptive immunity, leading to systemic immune dysregulation.^73^

## Role of balanced gut microbiota

A balanced microbiota in the gut is one that is diverse, stable, and beneficial to metabolic function. The gut microbiota produce various metabolites, such as lipopolysaccharide (LPS), short-chain fatty acids (SCFA) and trimethylamine-N-oxide, which play a vital role in communication between microbes and the host. Among the numerous roles that the gut microbiota performs are biodegradation of polysaccharides, maintaining the structural integrity of the gut mucosal barrier, immunomodulation, defending the host from pathogens and drug and nutrient metabolism.^2,75^

The small and large intestine possess an epithelial mucosal barrier to maintain a balance between beneficial and harmful microbes and their metabolites.^75,76^ Briefly, the mucus layer, with an outer section colonized by bacteria and an inner section devoid of bacteria, acts as a physical barrier. Composed of glycosylated mucin proteins, it separates gut microbes from direct epithelial contact.^77^ The intestinal epithelium consists of a monolayer of intestinal epithelial cells (IEC), primarily made up of enterocytes and specialized cells, which serve as the primary point of contact between the host and microbes.^78,79^ Enteroendocrine cells (EEC) produce hormones, goblet cells produce mucus, paneth cells secrete antimicrobial peptides and microfold cells secrete immunoglobulins A (IgA) into the inner mucus, providing protection against bacterial invasion.^80^ Transmembrane protein complexes, such as tight junctions and adherens junctions, that regulate intestinal permeability interconnect IEC.^81^

Tight junction proteins such as occludin and claudin interact with intracellular protein zonula occludens-1 (ZO-1) to form sealing strands for the paracellular gap between IECs and regulate selective passage of ions and solutes.^81^ Adherens junctions (e.g., E-cadherin) mediate cell-to-cell contact between IECs.^81^ IECs interact positively and negatively with gut microbiota to trigger immune cells to accept certain microbiota, maintaining enteric homeostasis.^76,78,82^ The lamina propria, a connective tissue layer, houses both innate and adaptive immune cells, including macrophages, dendritic cells, T-cells and B-cells within the Peyer’s patches.^78,80^ The presence of this epithelial-mucosal barrier enables the gut microbiota to contribute to physiological homeostasis by sustaining a symbiotic relationship with the host.

Gut microbiota are involved in the metabolism of carbohydrates, proteins, and to a lesser extent, lipids during digestion. Lipid metabolites are readily absorbed in the lower gastrointestinal system and contribute significantly to host energy production.^83^ Several bacteria including *Faecalibacterium prausnitzii*, and *Roseburia intestinalis*, ferment indigestible carbohydrates to produce SCFAs.^84^ SCFAs, primarily composed of acetate, propionate, and butyrate, primarily serve as an energy source for colonocytes, while the excess SCFAs can be transported to various tissues including the brain, heart, and lungs through the bloodstream. Although not fully understood, there is evidence that SCFAs play a vital role in a range of physiological processes including gut-barrier function, appetite regulation, downregulation of inflammation, blood pressure, and insulin sensitivity.^84–87^ For instance, SCFAs directly binding to G protein-coupled receptors on EECs triggers GLP-1 secretion, which enhances insulin sensitivity, central satiety, while also directly stimulating insulin secretion in pancreatic β-cells.^85^ Additionally, the gut microbiota synthesizes vitamins like K, B12, folic acid, and biotin, which the host cannot produce independently.^83,88^

## Role of gastrointestinal microbiota in the pathophysiology of obesity

Any disruptions to the gut microbiota equilibrium is called gut dysbiosis.^86^ Gut dysbiosis alters the intestinal barrier, and disrupts the crucial interactions between the host and microbiota, rendering the host more susceptible to obesity-related diseases.^86^ This disruption often involves reduced microbial diversity, leading to a decline in beneficial microbes and an increase in pathogenic microbes.^89^ An association between prolonged consumption of a western diet and gut dysbiosis in patients with obesity, leading to reduced microbial diversity and gene richness has been reported.^3^ Gut dysbiosis is further linked to increased intestinal permeability, adiposity, low-grade inflammation, dyslipidemia, and impaired glucose homeostasis.^90^

### Intestinal hyperpermeability

Accumulating evidence suggests that excessive consumption of dietary fat can result in increased permeability of the gut, commonly referred to as ‘leaky gut.’ Mice studies have shown that a high-fat diet (HFD) negatively affects the intestinal mucus layer, which becomes thinner and less effective in obesity.^91,92^ Excessive FFAs induce oxidative stress in goblet cells within the gut lumen, inhibiting mucus secretion and reducing growth rate and thus promotes thinning of the mucus layer, therefore allowing intestinal hyperpermeability.^91,93^

**Figure 3.**
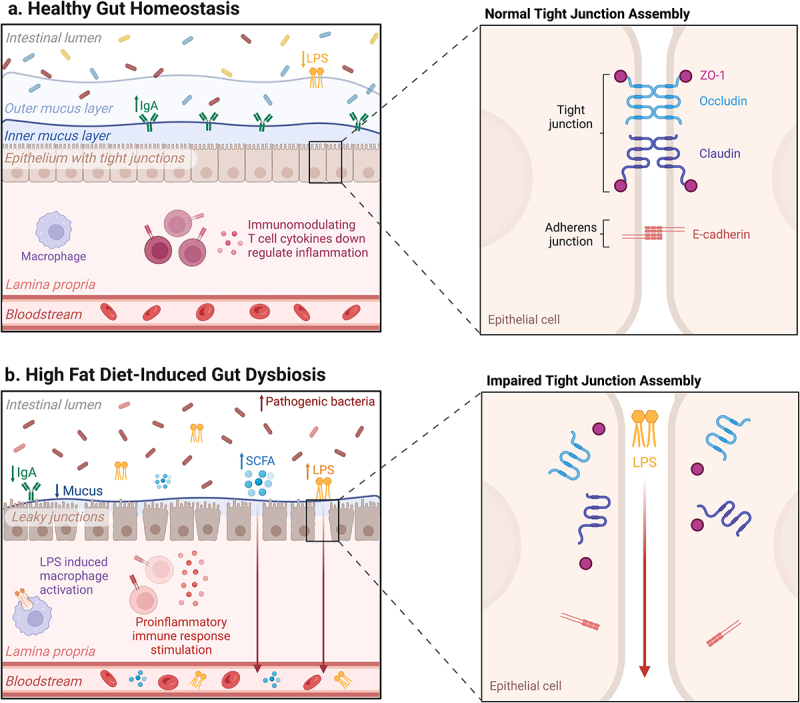
Comparison of the gastrointestinal landscape in a healthy state (a), compared to post-prolonged high-fat diet consumption (b): in a scenario of morbidly obese patients. High-fat diet alters the gut microbiota composition, diminishes the mucus layer, and damages the intestinal epithelial cells. This weakening of tight junctions and adherens junctions between intestinal cells leads to increased intestinal permeability. Consequently, bacterial metabolites like lipopolysaccharide (LPS) and short-chain fatty acids (SCFA) translocate from the gut lumen into the bloodstream, causing metabolic endotoxemia and excessive production of pro-inflammatory cytokines. Heightened cytokine levels and the interaction between bacterial metabolites and immune cells promote sustained inflammation of the intestinal tract. ZO-1, zonula occludens-1. IgA, immunoglobulins A. Created with BioRender.com.

Moreover, HFD is strongly linked to higher levels of plasma lipopolysaccharide (LPS) and increased Toll-like receptor (TLR) - 4.^94–97^ LPS are glycolipids in the cell wall of gram-negative bacteria which activate TLR-4 and trigger proinflammatory responses in various cells.^80^ Studies have shown that TLR-4 activation by LPS disrupts the tight junction proteins (e.g. occludins, claudins and ZO-1) and adherens junctions on the epithelial membrane, allowing LPS to enter the bloodstream.^80,98^ Through this proposed mechanism HFD dramatically downregulates the expression and distribution of tight junction-associated proteins, which help regulate intestinal permeability.^94,95,99^ In obese mice, the persistent elevation of pro-inflammatory cytokines (TNF-α, IL-6, and interferon-γ) released from inflamed adipose tissue or intestine have been found to disrupt tight junctions through stimulation of proinflammatory signaling pathways.^94^ These altered mechanisms collectively contribute to intestinal hyperpermeability or ‘leaky gut’ (Figure 3), allowing the passage of bacterial metabolites, food, and medication from the gut lumen into the bloodstream, triggering an immune response and promoting a pro-inflammatory state. However, the impact of a Western diet on intestinal barrier function is still uncertain and requires further investigation. Elevated levels of circulating LPS, known as metabolic endotoxemia, establish a cycle of immune system overstimulation, ultimately resulting in persistent, systemic inflammation.^80^ This ongoing inflammation further contributes to the development of obesity and associated health conditions.

Emerging evidence suggests that LPS plays a direct and indirect role in the obesity-induced changes in adipocytes. LPS triggers the transition of macrophages from the alternative M2 phenotype to the classical M1 phenotype by binding to TLR4 receptors on macrophage surfaces in adipocytes.^14,100,101^ M1 macrophages subsequently produce proinflammatory cytokines and ROS, promoting adiposity and inflammation.^11^ Another proposed inflammatory mechanism involves the uptake of LPS-rich lipoproteins by metabolically active hypertrophic adipocytes, leading to intracellular LPS-induced programmed adipocyte death (pyroptosis).^100,101^

LPS has been commonly used in animal models to induce vascular inflammation and endothelial dysfunction, although the precise underlying mechanism remains unclear.^102^ A cross-sectional study by Kim et al. demonstrated significantly higher LPS levels in hypertensive individuals compared to normotensive individuals.^103^ One possible explanation involves the activation of the oxidative TLR4-mediated pathway when exposed to LPS in vascular endothelial cells. This activation leads to the production of ROS, a decrease in nitric oxide availability, and consequently, endothelial dysfunction, contributing to an increase in blood pressure.^102,104,105^

Diet significantly influences the gut microbiota, impacting SCFA production, and contributing to its diverse effects. The association between SCFAs and obesity in both human and animal studies has yielded varying results, indicating a multifaceted and intricate role of SCFAs in obesity. Some studies have shown higher fecal SCFA concentrations in individuals with obesity compared to lean individuals, supporting the “energy harvesting” hypothesis.^106–109^ This hypothesis suggests that SCFAs serve as substrates for hepatic *de novo* lipogenesis, leading to increased fatty acid production and greater energy harvest from a HFD in an obese microbiome.^108,110,111^ Research by Goffredo et al. revealed that children with obesity had a greater capacity for fermenting carbohydrates, suggesting a higher presence of SCFA-producing bacteria in the microbiome of individuals with obesity.^112^ However, contrasting evidence has also emerged, (i.e., reduced SCFAs in obesity), suggesting an anti-obesity role.^98,113–117^ Supplementation of butyrate in obese mice increased insulin sensitivity and reduced adiposity, while acetate decreased lipid accumulation and inhibited adipocyte lipolysis.^118,119^ Additionally, individuals with obesity tend to have lower fasting levels of circulating GLP-1 and PYY compared to lean individuals.^120–124^ These hormones are released by SCFAs binding to intestinal GPCR on EECs, suppressing appetite, increasing satiety, and subsequently reducing food intake via gut-brain axis communication and delay gastric emptying.^125^ Overall, the relationship between SCFAs and obesity remains complex, and the underlying mechanisms of SCFAs’ actions are not fully understood, contributing to the ongoing interest in this research area.

### Altered abundance of Firmicutes and Bacteroidota in obesity

Firmicutes and Bacteroidota are the predominant bacterial phyla in the human gastrointestinal tract.^126^ The balance between these phyla, known as the F/B ratio, is crucial for maintaining homeostasis. Imbalances in this ratio can lead to various pathologies, including obesity.^127^ Using 16S rRNA gene sequencing, Ley and colleagues, found a 50% reduction in Bacteroidota and a significant increase in Firmicutes in genetically obese mice compared to their lean mice siblings.^128^ Turnbaugh et al. further confirmed that obese mice have a greater F/B ratio than lean mice using shotgun metagenomic sequencing of cecal microbial DNA.^110^ Similarly, a limited number of human studies have reported an elevated F/B ratio in individuals with obesity who consume a Western-style diet compared to lean individuals.^129–131^ It is hypothesized that Firmicutes are more efficient energy harvesters than Bacteroidota, resulting in more efficient calorie absorption and therefore weight gain.^132^ Specific Firmicutes species including *Ruminococcus bromii* are suggested to have the ability to produce more enzymes involved in carbohydrate degradation and fermentation than Bacteroidota species.^127^ The elevated F/B ratio may indicate a greater ability to ferment dietary polysaccharides into SCFAs, which aligns with the higher SCFAs levels observed in obesity.

A few studies have reported a lack of correlation between the F/B ratio and obesity, weight gain, or BMI.^133–135^ A study by Hu et al. revealed no differences in the F/B ratio of fecal samples from normal weight *versus* Korean adolescents with obesity at the phylum level.^136^ However, the study identified genus *Prevotella* (belonging to phylum Bacteroidota) to be more prevalent in subjects with obesity while genus *Bacteroides* (belonging to phylum Bacteroidota) was more prevalent in the lean group.^136^ To add to the inconsistencies, a few studies have reported decreased F/B ratios in individuals with obesity, favoring Bacteroidota.^101,137^ Interestingly, both Firmicutes and Bacteroidota phyla increased in a group with obesity *versus* healthy weight Egyptian subjects.^138^ These contradictory results could be attributed to a variety of factors, including different populations, age groups, gender, diet, cultural habit and genetics that play a role in differences found in gut microbiota composition. Although contradictions exist, most studies support the proposed explanation that Firmicutes possess a greater capacity for fermenting simple carbohydrates and metabolizing lipids, thereby effectively extracting more energy from HFD and contributing to the obesity epidemic.

### Beneficial gut bacteria decline and harmful bacteria rise in obesity

Gut dysbiosis is often characterized by an imbalance between beneficial bacteria and harmful bacteria. Obesity leads to a reduction in beneficial bacteria like *Akkermansia muciniphila* (Actinomycetota phylum). This reduction has been associated with increases in gut permeability and inflammation.^139,140^ This mucin-degrading bacterium resides in the mucus layer of the intestines and has beneficial effects on energy metabolism, fatty acid oxidation, down regulating inflammation and gut integrity.^139,141^ *A. muciniphila* decrease aligns with diminished mucus layer observed in obese mice. In a recent study by Mishra et al., it was found that obese mice exhibited a decrease in ethanolamine-metabolizing bacteria, resulting in elevated levels of ethanolamine, which promoted gut permeability by reducing the expression of the ZO-1 tight junction protein.^142^

Obesity not only reduces beneficial gut bacteria but also increases in bacteria positively correlated to inflammation. For instance, patients with obesity have shown increased levels of bacteria that exhibit inflammatory effects like *Staphylococcus spp*.^143^ Elevated Pseudomonadota levels have been reported in humans with obesity, which produce bacterial toxins such as LPS and spores that disrupt the gut epithelium, driving to metabolic endotoxemia and inflammation.^144–146^ Obesity also promotes the growth of pathogenic bacteria, including *Desulfovibrio spp*., which produces hydrogen sulfide gas causing apoptosis of IECs and increases intestinal permeability.^93,147^

Conversely, patients with obesity show a decreased abundance of certain bacteria like F. *prausnitzii* (phylum Firmicutes) and *Butyrivibrio* (phylum Bacillota), which are known to produce butyrate, a substance associated with anti-inflammatory properties.^148,149^ Butyrate enhances intestinal barrier function and mucosal immunity stimulating the secretion of IgA by plasma cells and anti-inflammatory cytokines by regulatory T cells, thereby reducing gut inflammation.^84,149^ Therefore, obesity-induced gut dysbiosis leads to an imbalance, favoring the overgrowth of potentially pathogenic bacteria while compromising beneficial bacteria, resulting in a chronic state of gut dysbiosis in individuals with obesity.

## Reversal of gut dysbiosis following bariatric surgery

Emerging scientific evidence indicates that restoring a well-balanced gut microbiome can benefit patients with obesity. Bariatric surgery has emerged as a potential catalyst for favorable shifts in gut microbiota composition, fostering a more balanced and diverse microbial community. However, this benefit is confined to the RYGB procedure as currently very limited studies have investigated the long term impact of OAGB on gut microbiota.

Compelling research suggests notable alterations in the gut microbiota of individuals with obesity before and after gastric bypass surgery. Initially, patients with obesity experience gut dysbiosis, characterized by decreased microbial diversity and richness, however, several studies have reported traditional RYGB to increase microbial diversity and richness with results persisting until 12 months post-surgery.^150–155^ The impact of OAGB on gut microbial diversity is less studied. A recent study conducted by Kaniel et al., investigating gut microbial composition after 6 months post-OAGB found a significant reduction in diversity compared to pre-surgery.^156^ These ongoing post-surgery microbiota changes reflect adaptations to anatomical and physiological modifications induced by the procedure, including reduced acid production, faster nutrient transit time, alterations in luminal pH, increased oxygen content, and changes in bile acid concentrations delivered to the colon.^157,158^ Further studies examining the impact of OAGB on the gut microbial diversity and richness over long-term are required.

Another relevant marker of gut dysbiosis in obese patients is proposed to be the F/B ratio, whereby a decreased ratio indicates restoration of the gut microbiota. Furet et al., demonstrated thirty patients with obesity had increased F/B ratio prior to RYGB, which reduced postoperatively at 3- and 6-months mark, concurrent with weight loss.^159^ As a result of gastric bypass surgery, it is theorized that since the number of calories and macronutrients consumed in food is considerably reduced, nutrient absorption is reduced since the duodenum and part of the jejunum is bypassed. A reduction of nutrient load affects the microbiota with Firmicutes declining and reduced capacity to accumulate fat and harvest energy from nutrients after gastric bypass leading to a rise in Bacteroidota.^150^ However, contradicting studies have not found any changes or reported decreased F/B ratio after RYGB.^132,153^ While the impact of OAGB on F/B ratio has yet to be studied, Mahmoudieh et al. found Firmicutes count significantly decreased 12 months post-OAGB in pooled samples of all patients across timepoints.^160^ This phenomenon can be attributed to the distinct anatomical alterations caused by OAGB as opposed to RYGB. These changes can influence the observed physiological variations resulting from surgery and may lead to gut microbial compositional shifts occurring at potentially lower taxonomic levels, such as the family, genus, or species level, which could be more relevant than the F/B ratio. Although the F/B ratio results vary across studies, they contribute to the growing body of evidence indicating that gastric bypass procedures lead to temporal and spatial alterations in the composition and function of the gut microbiota. It is important to note that the relationship between the F/B ratio and obesity is largely correlational, and this measure alone may not fully explain the microbial shifts occurring post-surgery. Further research should explore other taxonomic levels, such as genus or species, for a more nuanced understanding of microbial changes.

## Gut microbiota and metabolic changes after gastric bypass surgery

Bariatric surgery achieves improved metabolic status in patients with severe or morbid obesity which has been associated with gut microbial alterations. Traditional RYGB increased the abundance of beneficial species, including *A. muciniphila* 6-months post-surgery in fourteen patients with obesity.^161^ *A. muciniphila* is a known mucin degrader, and its increased abundance has been associated with improvement in glucose homeostasis and reduced adiposity in adults who are overweight and obese.^158^ In contrast, a recent study by Dao et al., reported no correlation between significantly increased abundance of *A. muciniphila* and glucose homeostasis 12-months post-RYGB in sixty-five females with severe obesity.^162^ However, considering the known beneficial role of *A. muciniphila* in increasing mucus thickness and maintaining gut epithelial barrier, this suggests that a particular level of *A. muciniphila* abundance may be necessary to observe major favorable health outcomes, such as improved gut barrier integrity and reduced inflammation.^163^ Furthermore, RYGB has been shown to elevate the presence of the potentially beneficial bacterium, *F. prausnitzii*, which is linked to reduced low-grade inflammation.^159,164^ However, it’s worth noting that contradictory findings also exist, with some studies reporting a decrease in the relative abundance of *F. prausnitzii* after RYGB in individuals with obesity.^150,165^ Graessler et al. reported *F. prausnitzii* decreased post-RYGB in parallel with inflammatory marker C-reactive protein.^165^ This decrease in abundance is unexpected as *F. prausnitzii* has been linked to beneficial effects on host metabolism, increased insulin sensitivity, and exhibits an anti-inflammatory effect.^165,166^ Therefore, alterations in abundances of beneficial gut bacteria following gastric bypass surgery imply a potentially positive impact on the host metabolic health and need to further studied especially in patients undergoing OAGB. However, these findings remain largely correlational, and more studies are needed to establish a causal relationship between these microbial changes and long-term metabolic outcomes.

Several animal and human studies have reported increased Pseudomonadota abundance following traditional RYGB surgery.^65,155,165–167^ This may be due to increased luminal pH, reduced gastric volume and greater transient oxygen exposure following gastric bypass surgery. A more alkaline environment encourages the proliferation of Pseudomonadota, which are less acid-tolerant than other phyla.^65^ Pseudomonadota has been associated with reduced systemic inflammation, weight loss and improved glycemic profile.^3,168^ In contradiction, a study of nine patients with morbid (class III) obesity, exhibited significantly reduced in Pseudomonadota abundance 15 months post-RYGB.^169^

A characteristic of gut dysbiosis is metabolic endotoxemia-induced chronic inflammation. In animal models, RYGB significantly reduced serum concentrations of LPS, inflammatory cytokines (IL6 and TNF-α), and intestinal permeability.^170–172^ Guo et al. revealed improved intestinal permeability in obese diabetic rats that underwent RYGB was positively correlated with LPS levels and negatively correlated with gut barrier proteins including ZO-1, occludin, and claudin-1 expression.^173^ The findings indicate that RYGB has the potential to diminish the severity of endotoxemia and inflammation, which correlates with enhanced tight junction integrity and overall intestinal barrier function. In humans, Troseid et al. found significantly reduced plasma LPS levels one year post-RYGB was directly correlated with HbA1c decrease, suggesting reduced LPS levels improve glycemic status in RYGB patients.^174^ Similarly, Monte et al. reported significantly reduced plasma LPS concentrations, TLR-4 expression, as well as inflammatory marker C-reactive protein at 6 months post-RYGB in fifteen adults with morbid obesity and T2DM.^175^ LPS-induced TLR-4 activation pathway leads to increased adiposity, excess pro-inflammatory and ROS production, as well as vascular endothelial dysfunction, raising blood pressure in patients with obesity. Reduced LPS levels in patients after gastric bypass induces a macrophage activation switch, favoring a shift toward M2 macrophages, over M1 macrophages in the adipose tissue.^176^ Theoretically, this would lead to reduced inflamed adipocytes, secretion of anti-inflammatory adipokines, subsequently, leading to improved insulin sensitivity.

Limited studies have investigated the effect of gastric bypass on the SCFA concentrations in patients with obesity. A recent systematic review reported significant reductions in SCFA levels following bariatric surgery in humans.^168^ Thirty individuals with obesity also experienced significant lower fecal SCFA levels 6 months post-RYGB.^177^ A possible explanation for SCFA decrease could be due to the diet modifications in patients who underwent bariatric surgery. Studies have reported decreased concentrations of total SCFAs after a high protein low-carbohydrate weight-loss diet in patients with obesity.^178,179^ SCFAs activate FFA receptors type 2 and 3 on immune cells and peripheral tissues including adipose tissue and pancreas, to trigger downstream pro-inflammatory signaling cascades.^87^ In contrast, RYGB has also been found to increase the formation of SCFAs in fourteen individuals with obesity 6 months post-surgery.^161^ Mukorako et al. demonstrated increased fecal SCFAs which were positively correlated with PYY hormone level post-RYGB in diet-induced obese rats.^180^ This association can be attributed to SCFAs directly modulating GLP-1 and PYY secretion. OAGB resulted in significantly higher serum GLP-1 levels in human and animal studies.^181,182^ Post-operative elevation of GLP-1 and PYY contributes to appetite suppression and promotes satiety by acting on the brain, inhibiting gastric motility and food intake.^183,184^ These hormones also improve glycemic control by stimulating insulin secretion, enhancing β-cell proliferation and inhibiting glucagon release from pancreatic islet α-cells.^183^

The existing literature primarily demonstrates correlational findings regarding the relationship between gut microbiota changes and metabolic outcomes following bariatric surgery. While these studies indicate significant shifts in microbiota composition post-surgery, it is essential to recognize that many of these associations lack established causal links. The heterogeneity of results in human studies may also stem from limited sample sizes, which often do not provide adequate statistical power to detect small variations in gut microbiota. The majority of these studies did not account for gender, along with lifestyle-related factors such as specific diets, food intake (pre-surgery versus long term post-surgery), antibiotics consumption, or physical activity, all of which could potentially influence the gut microbiota. Moreover, as the body adjusts to anatomical and physiological changes following bariatric surgery, it takes time to recover from the impact of a major surgery and achieve desirable outcomes. Because of this, short evaluation periods in most studies do not accurately reflect long-term changes in the gut microbiota following bariatric surgery. Despite these limitations, these studies contribute to the compelling evidence that gastric bypass bariatric surgery shifts the gut microbiota composition, potentially reversing gut dysbiosis. These alterations play an active role in achieving significant weight loss and resolving comorbidities in bariatric surgery patients with morbid obesity.

## Conclusion

Roux-en-Y Gastric Bypass (RYGB) has been extensively studied, demonstrating significant alterations in gut microbial composition that contribute to improved metabolic outcomes in patients with morbid obesity. Despite the growing adoption of OAGB among bariatric surgeons, research on its effects on gut microbiota remains notably limited. An ongoing clinical trial, the DIABAR-trial, is evaluating the benefits of laparoscopic RYGB and OAGB, including microbial, immunological, and metabolic markers, over a 10-year period in two-hundred twenty patients diagnosed with severe obesity and T2DM.^185^ However, there is a pressing need for more larger-scale studies, considering factors like diet, gender and age to comprehensively analyze gut microbiota changes post-RYGB and -OAGB over extensive periods. By identifying specific microbial patterns or species associated with the beneficial outcomes of these surgeries, researchers can better understand the mechanisms underlying metabolic improvements. This knowledge could pave way for innovative microbiota-based therapies, such as targeted probiotics, prebiotics, and fecal microbiota transplantation, which enhance the effectiveness of gastric bypass surgical outcomes by improving gut microbiota diversity and function, and ultimately reducing diet-induced obesity, MetS risk, and potential complications. In summary, understanding the impact of gastric bypass surgery impact on the gut microbiota is crucial for optimizing surgical benefits and advancing effective obesity treatments.

## Abbreviations

BMI: Body Mass Index
BP: Blood Pressure
CVD: Cardiovascular Disease
EEC: Enteroendocrine Cells
FFA: Free Fatty Acid
FMT: Fecal Microbiota Transplantation
GLP-1: Glucagon-like peptide-1
GLUT4: Glucose Transporter Type-4
HFD: High Fat Diet
HDL: High-Density Lipoprotein
IEC: Intestinal Epithelial Cell
IgA: Immunoglobulin A
IL-6: Interleukin-6
LDL: Low-Density Lipoprotein
LPS: Lipopolysaccharide
MetS: Metabolic Syndrome
MGB-OAGB: Mini Gastric Bypass-One Anastomosis Gastric Bypass
OAGB: One Anastomosis Gastric Bypass
PYY: Peptide YY
RAAS: Renin-Angiotensin-Aldosterone System
ROS: Reactive Oxygen Species
RYGB: Roux-en-Y Gastric Bypass
SCFA: Short-Chain Fatty Acid
SG: Sleeve Gastrectomy
SNS: Sympathetic Nervous System
T2DM: Type 2 Diabetes Mellitus
TGA: Therapeutic Drugs Administration
TLR: Toll-like Receptor
TNF-α: Tumor Necrosis Factor-Alpha
VLDL: Very-Low-Density Lipoprotein
ZO-1: Zonula occludens-1

## References

[1] Federico A, Dallio M, R DIS, Giorgio V, Miele L. Gut microbiota, obesity and metabolic disorders. Minerva Gastroenterol Dietol. 2017;63(4):337–24. doi:10.23736/S1121-421X.17.02376-5. 28927249

[2] Cheng Z, Zhang L, Yang L, Chu H. The critical role of gut microbiota in obesity. Front Endocrinol. 2022;13:1025706. doi:10.3389/fendo.2022.1025706. PMC9630587 36339448

[3] Debedat J, Clement K, Aron-Wisnewsky J. Gut microbiota dysbiosis in human obesity: impact of bariatric surgery. Curr Obes Rep. 2019 Sep. 8(3):229–242. doi:10.1007/s13679-019-00351-3. 31197613

[4] Clemente-Suarez VJ, Beltran-Velasco AI, Redondo-Florez L, Martin-Rodriguez A, Tornero-Aguilera JF. Global impacts of western diet and its effects on metabolism and health: a narrative review. Nutrients. 2023;15(12):2749. doi:10.3390/nu15122749. 37375654 PMC10302286

[5] Alberti KG, Eckel RH, Grundy SM, Zimmet PZ, Cleeman JI, Donato KA, Fruchart J-C, James WPT, Loria CM, Smith SC, et al. Harmonizing the metabolic syndrome: a joint interim statement of the international diabetes federation task force on epidemiology and prevention; national heart, lung, and blood institute; American heart association; world heart federation; international atherosclerosis society; and international association for the study of obesity. Circulation. 2009;120(16):1640–1645. doi:10.1161/CIRCULATIONAHA.109.192644. 19805654

[6] Ahmed B, Sultana R, Greene MW. Adipose tissue and insulin resistance in obese. Biomed Pharmacother. 2021;137:111315. doi:10.1016/j.biopha.2021.111315. 33561645

[7] Kawai T, Autieri MV, Scalia R. Adipose tissue inflammation and metabolic dysfunction in obesity. Am J Physiol Cell Physiol. 2021;320(3):C375–C391. doi:10.1152/ajpcell.00379.2020. 33356944 PMC8294624

[8] James DE, Stockli J, Birnbaum MJ. The aetiology and molecular landscape of insulin resistance. Nat Rev Mol Cell Biol. 2021;22(11):751–771. doi:10.1038/s41580-021-00390-6. 34285405

[9] Ormazabal V, Nair S, Elfeky O, Aguayo C, Salomon C, Zuniga FA. Association between insulin resistance and the development of cardiovascular disease. Cardiovasc Diabetol. 2018;17(1):122. doi:10.1186/s12933-018-0762-4. 30170598 PMC6119242

[10] Vekic J, Zeljkovic A, Stefanovic A, Jelic-Ivanovic Z, Spasojevic-Kalimanovska V. Obesity and dyslipidemia. Metabolism. 2019;92:71–81. doi:10.1016/j.metabol.2018.11.005. 30447223

[11] Shariq OA, McKenzie TJ. Obesity-related hypertension: a review of pathophysiology, management, and the role of metabolic surgery. Gland Surg. 2020;9(1):80–93. doi:10.21037/gs.2019.12.03. 32206601 PMC7082272

[12] Li Y, Li Z, Ngandiri DA, Llerins Perez M, Wolf A, Wang Y. The molecular brakes of adipose tissue lipolysis. Front Physiol. 2022;13:826314. doi:10.3389/fphys.2022.826314. 35283787 PMC8907745

[13] Leon-Pedroza JI, Gonzalez-Tapia LA, Del Olmo-Gil E, Castellanos-Rodriguez D, Escobedo G, Gonzalez-Chavez A. Low-grade systemic inflammation and the development of metabolic diseases: from the molecular evidence to the clinical practice. Cirugía y Cirujanos (Engl Ed). 2015 [Accessed 2023 Sep 6]. 83(6):543–551. https://www.sciencedirect.com/science/article/pii/S0009741115001188?via%3Dihub. 10.1016/j.circir.2015.05.041 26159364

[14] Odegaard JI, Chawla A. Alternative macrophage activation and metabolism. Annu Rev Pathol. 2011;6(1):275–297. doi:10.1146/annurev-pathol-011110-130138. 21034223 PMC3381938

[15] Achari AE, Jain SK. Adiponectin, a therapeutic target for obesity, diabetes, and endothelial dysfunction. Int J Mol Sci. 2017;18(6):1321. doi:10.3390/ijms18061321. 28635626 PMC5486142

[16] Hudish LI, Reusch JE, Sussel L. Beta cell dysfunction during progression of metabolic syndrome to type 2 diabetes. J Clin Invest. 2019;129(10):4001–4008. doi:10.1172/JCI129188. 31424428 PMC6763241

[17] Riddy DM, Delerive P, Summers RJ, Sexton PM, Langmead CJ. G protein-coupled receptors targeting insulin resistance, obesity, and type 2 diabetes mellitus. Pharmacol Rev. 2018;70(1):39–67. doi:10.1124/pr.117.014373. 29233848

[18] Ipsen DH, Tveden-Nyborg P, Lykkesfeldt J. Dyslipidemia: obese or not obese-that is not the question. Curr Obes Rep. 2016;5(4):405–412. doi:10.1007/s13679-016-0232-9. 27687811

[19] Bhat H, Bhat JA, Bhat MH, Rashid M, Jan R, Afroze D. Leptin in obesity and hypertension. Arterial Hypertens. 2022;26(1):26–31. doi:10.5603/ah.a2022.0003.

[20] Khodamoradi K, Khosravizadeh Z, Seetharam D, Mallepalli S, Farber N, Arora H. The role of leptin and low testosterone in obesity. Int J Impot Res. 2022;34(7):704–713. doi:10.1038/s41443-022-00534-y. 35102263

[21] Russo B, Menduni M, Borboni P, Picconi F, Frontoni S. Autonomic nervous system in obesity and insulin-Resistance—The complex interplay between leptin and central nervous system. Int J Mol Sci. 2021;22(10):5187. doi:10.3390/ijms22105187. 34068919 PMC8156658

[22] Obradovic M, Sudar-Milovanovic E, Soskic S, Essack M, Arya S, Stewart AJ, Gojobori T, Isenovic ER. Leptin and obesity: role and clinical implication. Front Endocrinol. 2021;12:585887. doi:10.3389/fendo.2021.585887. PMC8167040 34084149

[23] Fahed G, Aoun L, Bou Zerdan M, Allam S, Bou Zerdan M, Bouferraa Y, Assi HI. Metabolic syndrome: updates on pathophysiology and management in 2021. IJMS. 2022;23(2):786. doi:10.3390/ijms23020786. 35054972 PMC8775991

[24] Hall JE, Do Carmo Jm, da Silva Aa, Wang Z, Hall ME, Do Carmo JM, da Silva AA. Obesity-induced hypertension: interaction of neurohumoral and renal mechanisms. Circ Res. 2015;116(6):991–1006. doi:10.1161/CIRCRESAHA.116.305697. 25767285 PMC4363087

[25] Thethi T, Kamiyama M, Kobori H. The link between the renin-angiotensin-aldosterone system and renal injury in obesity and the metabolic syndrome. Curr Hypertens Rep. 2012;14(2):160–169. doi:10.1007/s11906-012-0245-z. 22302531 PMC3337881

[26] Kwaifa IK, Bahari H, Yong YK, Noor SM. Endothelial dysfunction in obesity-induced inflammation: molecular mechanisms and clinical implications. Biomolecules. 2020;10(2):291. doi:10.3390/biom10020291. 32069832 PMC7072669

[27] Yao L, Herlea-Pana O, Heuser-Baker J, Chen Y, Barlic-Dicen J. Roles of the chemokine system in development of obesity, insulin resistance, and cardiovascular disease. J Immunol Res. 2014;2014:181450. doi:10.1155/2014/181450. 24741577 PMC3987870

[28] Saris WH, Blair SN, van Baak MA, Eaton SB, Davies PSW, Di Pietro L, Fogelholm M, Rissanen A, Schoeller D, Swinburn B, et al. How much physical activity is enough to prevent unhealthy weight gain? Outcome of the iaso 1^st^ stock conference and consensus statement. Obes Rev. 2003;4(2):101–114. doi:10.1046/j.1467-789x.2003.00101.x. 12760445

[29] Coulter AA, Rebello CJ, Greenway FL. Centrally acting agents for obesity: past, present, and future. Drugs. 2018;78(11):1113–1132. doi:10.1007/s40265-018-0946-y. 30014268 PMC6095132

[30] Ard J, Fitch A, Fruh S, Herman L. Weight loss and maintenance related to the mechanism of action of glucagon-like peptide 1 receptor agonists. Adv Ther. 2021;38(6):2821–2839. doi:10.1007/s12325-021-01710-0. 33977495 PMC8189979

[31] Billes SK, Sinnayah P, Cowley MA. Naltrexone/Bupropion for obesity: an investigational combination pharmacotherapy for weight loss. Pharmacol Res. 2014;84:1–11. doi:10.1016/j.phrs.2014.04.004. 24754973

[32] Son JW, Kim S. Comprehensive review of current and upcoming anti-obesity drugs. Diabetes Metab J. 2020;44(6):802–818. doi:10.4093/dmj.2020.0258. 33389955 PMC7801751

[33] Walmsley R, Sumithran P. Current and emerging medications for the management of obesity in adults. Med J Aust. 2023;219(4):188. doi:10.5694/mja2.52031. 37402483

[34] Lenz TL, Hamilton WR. Supplemental products used for weight loss. J Am Pharm Assoc. 2004;44(1):59–67; quiz 67–8. doi:10.1331/154434504322713246. 14965155

[35] Heymsfield SB, van Mierlo Ca, van der Knaap Hc, Heo M, Frier HI, van Mierlo CAJ, van der Knaap HCM. Weight management using a meal replacement strategy: meta and pooling analysis from six studies. Int J Obes Relat Metab Disord. 2003;27(5):537–549. doi:10.1038/sj.ijo.0802258. 12704397

[36] Ard JD, Lewis KH, Rothberg A, Auriemma A, Coburn SL, Cohen SS, Loper J, Matarese L, Pories WJ, Periman S, et al. Effectiveness of a total meal replacement program (OPTIFAST program) on weight loss: results from the OPTIWIN study. Obesity. 2019;27(1):22–29. doi:10.1002/oby.22303. 30421863 PMC6587830

[37] Oster M, Hein N, Aksan A, Krammer H, Theodoridou S, Stein J. Efficacy and safety of intragastric balloon therapy compared to a multidisciplinary weight loss program (optifast) in a real-world population: a propensity score matching analysis. Obes Facts. 2023;16(1):89–98. doi:10.1159/000524895. 36257288 PMC9889727

[38] Eisenberg D, Shikora SA, Aarts E, Aminian A, Angrisani L, Cohen RV, de Luca M, Faria SL, Goodpaster KPS, Haddad A, et al. 2022 American society of metabolic and bariatric surgery (ASMBS) and international federation for the surgery of obesity and metabolic disorders (IFSO) indications for metabolic and bariatric surgery. Obes Surg. 2023; 2023;33(1):3–14. doi:10.1007/s11695-022-06332-1. 36336720 PMC9834364

[39] Arterburn D, Wellman R, Emiliano A, Smith SR, Odegaard AO, Murali S, Williams N, Coleman KJ, Courcoulas A, Coley RY, et al. Comparative effectiveness and safety of bariatric procedures for weight loss: a PCORnet cohort study. Ann Intern Med. 2018;169(11):741–750. doi:10.7326/M17-2786. 30383139 PMC6652193

[40] Mason EE, Ito C. Gastric bypass in obesity. Surg Clin N Am. 1967;47(6):1345–1351. doi:10.1016/s0039-6109(16)38384-0. 6073761

[41] Mitchell BG, Gupta N. Roux-en-Y Gastric Bypass. StatPearls. 2023 [Accessed 2023 Aug 6]. https://pubmed.ncbi.nlm.nih.gov/31985950/.

[42] Rutledge R. The mini-gastric bypass: experience with the first 1,274 cases. Obes Surg. 2001;11(3):276–280. doi:10.1381/096089201321336584. 11433900

[43] Carbajo M, Garcia-Caballero M, Toledano M, Osorio D, Garcia-Lanza C, Carmona JA. One-anastomosis gastric bypass by laparoscopy: results of the first 209 patients. Obes Surg. 2005;15(3):398–404. doi:10.1381/0960892053576677. 15826476

[44] Deitel M. History of the MGB and OAGB operations. Int J Surg. 2019;66:79–83. doi:10.1016/j.ijsu.2019.04.018. 31054329

[45] De Luca M, Tie T, Ooi G, Higa K, Himpens J, Carbajo M-A, Mahawar K, Shikora S, Brown WA. Mini gastric bypass-one anastomosis gastric bypass (MGB-OAGB)-ifso position statement. Obes Surg. 2018;28(5):1188–1206. doi:10.1007/s11695-018-3182-3. 29600339

[46] Kruschitz R, Wakolbinger M, Schindler K, Prager G, Hoppichler F, Marculescu R, Ludvik B. Effect of one-anastomosis gastric bypass on cardiovascular risk factors in patients with vitamin d deficiency and morbid obesity: a secondary analysis. Nutr Metab Cardiovasc Dis. 2020;30(12):2379–2388. doi:10.1016/j.numecd.2020.08.011. 32981799

[47] Kessler Y, Adelson D, Mardy-Tilbor L, Ben-Porat T, Szold A, Goitein D, Sakran N, Raziel A, Sherf-Dagan S. Nutritional status following one anastomosis gastric bypass. Clin Nutr. 2020;39(2):599–605. doi:10.1016/j.clnu.2019.03.008. 30922792

[48] Bettini S, Segato G, Prevedello L, Fabris R, Prà CD, Zabeo E, Compagnin C, De Luca F, Finco C, Foletto M, et al. Improvement of lipid profile after one-anastomosis gastric bypass compared to sleeve gastrectomy. Nutrients. 2021;13(8):2770. doi:10.3390/nu13082770. 34444930 PMC8401377

[49] Robert M, Espalieu P, Pelascini E, Caiazzo R, Sterkers A, Khamphommala L, Poghosyan T, Chevallier J-M, Malherbe V, Chouillard E, et al. Efficacy and safety of one anastomosis gastric bypass versus roux-en-Y gastric bypass for obesity (YOMEGA): a multicentre, randomised, open-label, non-inferiority trial. The Lancet. 2019;393(10178):1299–1309. doi:10.1016/S0140-6736(19)30475-1. 30851879

[50] Singh B, Saikaustubh Y, Singla V, Kumar A, Ahuja V, Gupta Y, Kashyap L, Aggarwal S. One anastomosis gastric bypass (OAGB) vs roux-en-y gastric bypass (RYGB) for remission of T2DM in patients with morbid obesity: a randomized controlled trial. Obes Surg. 2023;33(4):1218–1227. doi:10.1007/s11695-023-06515-4. 36807043

[51] Rheinwalt KP, Plamper A, Ruckbeil MV, Kroh A, Neumann UP, Ulmer TF. One anastomosis gastric bypass-mini-gastric bypass (OAGB-MGB) versus roux-en-y gastric bypass (RYGB)-a mid-term cohort study with 612 patients. Obes Surg. 2020;30(4):1230–1240. doi:10.1007/s11695-019-04250-3. 31758474

[52] Level L, Rojas A, Pinango S, Avariano Y. One anastomosis gastric bypass vs. roux-en-y gastric bypass: a 5-year follow-up prospective randomized trial. Langenbecks Arch Surg. 2021;406(1):171–179. doi:10.1007/s00423-020-01949-1. 32761373

[53] Bhandari M, Nautiyal HK, Kosta S, Mathur W, Fobi M. Comparison of one-anastomosis gastric bypass and roux-en-y gastric bypass for treatment of obesity: a 5-year study. Surg Obes Relat Dis. 2019;15(12):2038–2044. doi:10.1016/j.soard.2019.05.025. 31734066

[54] Kermansaravi M, Shahmiri SS, DavarpanahJazi AH, Valizadeh R, Berardi G, Vitiello A, Musella M, Carbajo M. One anastomosis/mini-gastric bypass (OAGB/MGB) as revisional surgery following primary restrictive bariatric procedures: a systematic review and meta-analysis. Obes Surg. 2021;31(1):370–383. doi:10.1007/s11695-020-05079-x. 33118133 PMC7809003

[55] Parmar CD, Gan J, Stier C, Dong Z, Chiappetta S, El-Kadre L, Bashah MM, Wang C, Sakran N. One anastomosis/mini gastric bypass (OAGB-MGB) as revisional bariatric surgery after failed primary adjustable gastric band (LAGB) and sleeve gastrectomy (SG): a systematic review of 1075 patients. Int J Surg. 2020;81:32–38. doi:10.1016/j.ijsu.2020.07.007. 32738545

[56] Poublon N, Chidi I, Bethlehem M, Kuipers E, Gadiot R, Emous M, van Det M, Dunkelgrun M, Biter U, Apers J, et al. One anastomosis gastric bypass vs. roux-en-y gastric bypass, remedy for insufficient weight loss and weight regain after failed restrictive bariatric surgery. Obes Surg. 2020;30(9):3287–3294. doi:10.1007/s11695-020-04536-x. 32307669 PMC7378100

[57] Parikh M, Eisenberg D, Johnson J, El-Chaar M. American society for m, bariatric surgery clinical issues c. American society for metabolic and bariatric surgery review of the literature on one-anastomosis gastric bypass. Surg Obes Relat Dis. 2018;14(8):1088–1092. doi:10.1016/j.soard.2018.04.017. 29907540

[58] Genser L, Carandina S, Tabbara M, Torcivia A, Soprani A, Siksik J-M, Cady J. Presentation and surgical management of leaks after mini–gastric bypass for morbid obesity. Surg Obes Relat Dis. 2016;12(2):305–312. doi:10.1016/j.soard.2015.06.010. 26410539

[59] Keleidari B, Dehkordi MM, Shahraki MS, Ahmadi ZS, Heidari M, Hajian A, Nasaj HT. Bile reflux after one anastomosis gastric bypass surgery: a review study. Ann Med Surg (Lond). 2021;64:102248. doi:10.1016/j.amsu.2021.102248. 33868682 PMC8040101

[60] Keleidari B, Mahmoudieh M, Davarpanah Jazi AH, Melali H, Nasr Esfahani F, Minakari M, Mokhtari M. Comparison of the bile reflux frequency in one anastomosis gastric bypass and roux-en-y gastric bypass: a cohort study. Obes Surg. 2019;29(6):1721–1725. doi:10.1007/s11695-018-03683-6. 30767188

[61] Aleman R, Lo Menzo E, Szomstein S, Rosenthal RJ. Efficiency and risks of one-anastomosis gastric bypass. Ann Transl Med. 2020;8(Suppl 1):S7. doi:10.21037/atm.2020.02.03. 32309411 PMC7154323

[62] Solouki A, Kermansaravi M, Davarpanah Jazi AH, Kabir A, Farsani TM, Pazouki A. One-anastomosis gastric bypass as an alternative procedure of choice in morbidly obese patients. J Res Med Sci. 2018;23(1):84. doi:10.4103/jrms.JRMS_386_18. 30294352 PMC6161487

[63] Lee WJ, Lee YC, Ser KH, Chen SC, Chen JC, Su YH. Revisional surgery for laparoscopic mini gastric bypass. Surg Obes Relat Dis. 2011;7(4):486–491. doi:10.1016/j.soard.2010.10.012. 21159561

[64] Quercia I, Dutia R, Kotler DP, Belsley S, Laferrere B. Gastrointestinal changes after bariatric surgery. Diabetes Metab. 2014;40(2):87–94. doi:10.1016/j.diabet.2013.11.003. 24359701 PMC4391395

[65] Dang JT, Mocanu V, Park H, Laffin M, Hotte N, Karmali S, Birch DW, Madsen KL. Roux-en-y gastric bypass and sleeve gastrectomy induce substantial and persistent changes in microbial communities and metabolic pathways. Gut Microbes. 2022;14(1):2050636. doi:10.1080/19490976.2022.2050636. 35316158 PMC8942407

[66] Ashrafian H, le Roux CW, Rowland SP, Ali M, Cummin AR, Darzi A, Athanasiou T. Metabolic surgery and obstructive sleep apnoea: the protective effects of bariatric procedures. Thorax. 2012;67(5):442–449. doi:10.1136/thx.2010.151225. 21709167

[67] Arterburn DE, Telem DA, Kushner RF, Courcoulas AP. Benefits and risks of bariatric surgery in adults: a review. JAMA. 2020;324(9):879–887. doi:10.1001/jama.2020.12567. 32870301

[68] D’Argenio V, Salvatore F. The role of the gut microbiome in the healthy adult status. Clin Chim Acta. 2015;451(Pt A):97–102. doi:10.1016/j.cca.2015.01.003. 25584460

[69] Wen L, Duffy A. Factors influencing the gut microbiota, inflammation, and type 2 diabetes. J Nutr. 2017;147(7):1468S–1475S. doi:10.3945/jn.116.240754. 28615382 PMC5483960

[70] Niu J, Xu L, Qian Y, Sun Z, Yu D, Huang J, Zhou X, Wang Y, Zhang T, Ren R, et al. Evolution of the gut microbiome in early childhood: a cross-sectional study of Chinese children. Front Microbiol. 2020;11:439. doi:10.3389/fmicb.2020.00439. 32346375 PMC7169428

[71] Eckburg PB, Bik EM, Bernstein CN, Purdom E, Dethlefsen L, Sargent M, Gill SR, Nelson KE, Relman DA. Diversity of the human intestinal microbial flora. Science. 2005;308(5728):1635–1638. doi:10.1126/science.1110591. 15831718 PMC1395357

[72] Ley RE, Turnbaugh PJ, Klein S, Gordon JI. Microbial ecology: human gut microbes associated with obesity. Nature. 2006;444(7122):1022–1023. doi:10.1038/4441022a. 17183309

[73] Zhao T, Wei Y, Zhu Y, Xie Z, Hai Q, Li Z, Qin D. Gut microbiota and rheumatoid arthritis: from pathogenesis to novel therapeutic opportunities. Front Immunol. 2022;13:1007165. doi:10.3389/fimmu.2022.1007165. 36159786 PMC9499173

[74] Dehner C, Fine R, Kriegel MA. The microbiome in systemic autoimmune disease: mechanistic insights from recent studies. Curr Opin Rheumatol. 2019;31(2):201–207. doi:10.1097/BOR.0000000000000574. 30624285 PMC6408954

[75] Jandhyala SM, Talukdar R, Subramanyam C, Vuyyuru H, Sasikala M, Nageshwar Reddy D. Role of the normal gut microbiota. World J Gastroenterol. 2015;21(29):8787–8803. doi:10.3748/wjg.v21.i29.8787. 26269668 PMC4528021

[76] Ruth MR, Field CJ. The immune modifying effects of amino acids on gut-associated lymphoid tissue. J Anim Sci Biotechnol. 2013;4(1):27. doi:10.1186/2049-1891-4-27. 23899038 PMC3750756

[77] Okumura R, Takeda K. Roles of intestinal epithelial cells in the maintenance of gut homeostasis. Exp Mol Med. 2017;49(5):e338. doi:10.1038/emm.2017.20. 28546564 PMC5454438

[78] Sharma L, Riva A. Intestinal barrier function in health and disease—any role of SARS-CoV-2? Microorganisms. 2020;8(11):1744. doi:10.3390/microorganisms8111744. 33172188 PMC7694956

[79] Sterling KG, Dodd GK, Alhamdi S, Asimenios PG, Dagda RK, De Meirleir KL, Hudig D, Lombardi VC. Mucosal immunity and the gut-microbiota-brain-axis in neuroimmune disease. Int J Mol Sci. 2022;23(21):13328. doi:10.3390/ijms232113328. 36362150 PMC9655506

[80] Mohammad S, Thiemermann C. Role of metabolic endotoxemia in systemic inflammation and potential interventions. Front Immunol. 2020;11:594150. doi:10.3389/fimmu.2020.594150. 33505393 PMC7829348

[81] Hartsock A, Nelson WJ. Adherens and tight junctions: structure, function and connections to the actin cytoskeleton. Biochim Biophys Acta. 2008;1778(3):660–669. doi:10.1016/j.bbamem.2007.07.012. 17854762 PMC2682436

[82] Chelakkot C, Ghim J, Ryu SH. Mechanisms regulating intestinal barrier integrity and its pathological implications. Exp Mol Med. 2018;50(8):1–9. doi:10.1038/s12276-018-0126-x. PMC6095905 30115904

[83] Oliphant K, Allen-Vercoe E. Macronutrient metabolism by the human gut microbiome: major fermentation by-products and their impact on host health. Microbiome. 2019;7(1):91. doi:10.1186/s40168-019-0704-8. 31196177 PMC6567490

[84] Parada Venegas D, la Fuente Mk D, Landskron G, González MJ, Quera R, Dijkstra G, Harmsen HJM, Faber KN, Hermoso MA. Short chain fatty acids (SCFAs)-mediated gut epithelial and immune regulation and its relevance for inflammatory bowel diseases. Front Immunol. 2019;10:277. doi:10.3389/fimmu.2019.00277. 30915065 PMC6421268

[85] la Cuesta-Zuluaga J D, Mueller NT, Alvarez-Quintero R, Velásquez-Mejía E, Sierra J, Corrales-Agudelo V, Carmona J, Abad J, Escobar J. Higher fecal short-chain fatty acid levels are associated with gut microbiome dysbiosis, obesity, hypertension and cardiometabolic disease risk factors. Nutrients. 2018;11(1):51. doi:10.3390/nu11010051. 30591685 PMC6356834

[86] Yoo JY, Groer M, Dutra SVO, Sarkar A, McSkimming DI. Gut microbiota and immune system interactions. Microorganisms. 2020;8(10):1587. doi:10.3390/microorganisms8101587. 33076307 PMC7602490

[87] Li M, van Esch B, Wagenaar GTM, Garssen J, Folkerts G, Henricks PAJ. Pro- and anti-inflammatory effects of short chain fatty acids on immune and endothelial cells. Eur J Pharmacol. 2018;831:52–59. doi:10.1016/j.ejphar.2018.05.003. 29750914

[88] Tremaroli V, Bäckhed F. Functional interactions between the gut microbiota and host metabolism. Nature. 2012;489(7415):242–249. doi:10.1038/nature11552. 22972297

[89] DeGruttola AK, Low D, Mizoguchi A, Mizoguchi E. Current understanding of dysbiosis in disease in human and animal models. Inflamm Bowel Dis. 2016;22(5):1137–1150. doi:10.1097/MIB.0000000000000750. 27070911 PMC4838534

[90] Le Chatelier E, Nielsen T, Qin J, Prifti E, Hildebrand F, Falony G, Almeida M, Arumugam M, Batto J-M, Kennedy S, et al. Richness of human gut microbiome correlates with metabolic markers. Nature. 2013;500(7464):541–546. doi:10.1038/nature12506. 23985870

[91] Schroeder BO, Birchenough GMH, Pradhan M, Nyström EEL, Henricsson M, Hansson GC, Bäckhed F. Obesity-associated microbiota contributes to mucus layer defects in genetically obese mice. J Biol Chem. 2020;295(46):15712–15726. doi:10.1074/jbc.RA120.015771. 32900852 PMC7667970

[92] Mukai R, Handa O, Naito Y, Takayama S, Suyama Y, Ushiroda C, Majima A, Hirai Y, Mizushima K, Okayama T, et al. High-fat diet causes constipation in mice via decreasing colonic mucus. Dig Dis Sci. 2020;65(8):2246–2253. doi:10.1007/s10620-019-05954-3. 31728788

[93] Rohr MW, Narasimhulu CA, Rudeski-Rohr TA, Parthasarathy S. Negative effects of a high-fat diet on intestinal permeability: a review. Adv Nutr. 2020;11(1):77–91. doi:10.1093/advances/nmz061. 31268137 PMC7442371

[94] Kim KA, Gu W, Lee IA, Joh EH, Kim DH, Chamaillard M. High fat diet-induced gut microbiota exacerbates inflammation and obesity in mice via the TLR4 signaling pathway. PLoS One. 2012;7(10):e47713. doi:10.1371/journal.pone.0047713. 23091640 PMC3473013

[95] Cani PD, Bibiloni R, Knauf C, Waget A, Neyrinck AM, Delzenne NM, Burcelin R. Changes in gut microbiota control metabolic endotoxemia-induced inflammation in high-fat diet–induced obesity and diabetes in mice. Diabetes. 2008;57(6):1470–1481. doi:10.2337/db07-1403. 18305141

[96] Ghanim H, Abuaysheh S, Sia CL, Korzeniewski K, Chaudhuri A, Fernandez-Real JM, Dandona P. Increase in plasma endotoxin concentrations and the expression of Toll-like receptors and suppressor of cytokine signaling-3 in mononuclear cells after a high-fat, high-carbohydrate meal: implications for insulin resistance. Diabetes Care. 2009;32(12):2281–2287. doi:10.2337/dc09-0979. 19755625 PMC2782991

[97] de La Serre Cb, Ellis CL, Lee J, Hartman AL, Rutledge JC, Raybould HE, de La Serre CB. Propensity to high-fat diet-induced obesity in rats is associated with changes in the gut microbiota and gut inflammation. Am J Physiol Gastrointest Liver Physiol. 2010;299(2):G440–8. doi:10.1152/ajpgi.00098.2010. 20508158 PMC2928532

[98] Song B, Zhao K, Zhou S, Xue Y, Lu H, Jia X, Wang S. Association of the gut microbiome with fecal short-chain fatty acids, lipopolysaccharides, and obesity in young Chinese college students. Front Nutr. 2023;10:1057759. doi:10.3389/fnut.2023.1057759. 37139436 PMC10150786

[99] Usuda H, Okamoto T, Wada K. Leaky gut: effect of dietary fiber and fats on microbiome and intestinal barrier. Int J Mol Sci. 2021;22(14):7613. doi:10.3390/ijms22147613. 34299233 PMC8305009

[100] Hersoug LG, Moller P, Loft S. Role of microbiota-derived lipopolysaccharide in adipose tissue inflammation, adipocyte size and pyroptosis during obesity. Nutr Res Rev. 2018 Dec. 31(2):153–163. doi:10.1017/S0954422417000269. 29362018

[101] Hersoug LG, Moller P, Loft S. Gut microbiota-derived lipopolysaccharide uptake and trafficking to adipose tissue: implications for inflammation and obesity. Obes Rev. 2016 Apr. 17(4):297–312. doi:10.1111/obr.12370. 26712364

[102] Grylls A, Seidler K, Neil J. Link between microbiota and hypertension: focus on LPS/TLR4 pathway in endothelial dysfunction and vascular inflammation, and therapeutic implication of probiotics. Biomed Pharmacother. 2021;137:111334. doi:10.1016/j.biopha.2021.111334. 33556874

[103] Kim S, Goel R, Kumar A, Qi Y, Lobaton G, Hosaka K, Mohammed M, Handberg E, Richards E, Pepine C, et al. Imbalance of gut microbiome and intestinal epithelial barrier dysfunction in patients with high blood pressure. Clin Sci. 2018;132(6):701–718. doi:10.1042/CS20180087. PMC5955695 29507058

[104] Dauphinee SM, Karsan A. Lipopolysaccharide signaling in endothelial cells. Lab Invest. 2006;86(1):9–22. doi:10.1038/labinvest.3700366/. 16357866

[105] Munshi N, Fernandis AZ, Cherla RP, Park IW, Ganju RK. Lipopolysaccharide-induced apoptosis of endothelial cells and its inhibition by vascular endothelial growth factor. J Immunol. 2002;168(11):5860–5866. doi:10.4049/jimmunol.168.11.5860/. 12023390

[106] Rahat-Rozenbloom S, Fernandes J, Gloor GB, Wolever TM. Evidence for greater production of colonic short-chain fatty acids in overweight than lean humans. Int J Obes. 2014;38(12):1525–1531. doi:10.1038/ijo.2014.46. PMC3970979 24642959

[107] Schwiertz A, Taras D, Schafer K, Beijer S, Bos NA, Donus C, Hardt PD. Microbiota and SCFA in lean and overweight healthy subjects. Obesity. 2010;18(1):190–195. doi:10.1038/oby.2009.167. 19498350

[108] Fernandes J, Su W, Rahat-Rozenbloom S, Wolever TM, Comelli EM. Adiposity, gut microbiota and faecal short chain fatty acids are linked in adult humans. Nutr & Diabetes. 2014;4(6):e121. doi:10.1038/nutd.2014.23. PMC4079931 24979150

[109] Teixeira TF, Grzeskowiak L, Franceschini SC, Bressan J, Ferreira CL, Peluzio MC. Higher level of faecal SCFA in women correlates with metabolic syndrome risk factors. Br J Nutr. 2013;109(5):914–919. doi:10.1017/S0007114512002723. 23200109

[110] Turnbaugh PJ, Ley RE, Mahowald MA, Magrini V, Mardis ER, Gordon JI. An obesity-associated gut microbiome with increased capacity for energy harvest. Nature. 2006;444(7122):1027–1031. doi:10.1038/nature05414. 17183312

[111] Turnbaugh PJ, Backhed F, Fulton L, Gordon JI. Diet-induced obesity is linked to marked but reversible alterations in the mouse distal gut microbiome. Cell Host & Microbe. 2008;3(4):213–223. doi:10.1016/j.chom.2008.02.015. 18407065 PMC3687783

[112] Goffredo M, Mass K, Parks EJ, Wagner DA, McClure EA, Graf J, Savoye M, Pierpont B, Cline G, Santoro N, et al. Role of gut microbiota and short chain fatty acids in modulating energy harvest and fat partitioning in youth. J Clin Endocrinol Metab. 2016;101(11):4367–4376. doi:10.1210/jc.2016-1797. 27648960 PMC5095239

[113] Barczynska R, Litwin M, Slizewska K, Szalecki M, Berdowska A, Bandurska K, Libudzisz Z, Kapuśniak J. Bacterial microbiota and fatty acids in the faeces of overweight and obese children. Pol J Microbiol. 2018;67(3):339–345. doi:10.21307/pjm-2018-041. 30451451 PMC7256813

[114] den Besten G, Bleeker A, Gerding A, den Besten G, van Eunen K, Havinga R, van Dijk TH, Oosterveer MH, Jonker JW, Groen AK, et al. Short-chain fatty acids protect against high-fat diet–induced obesity via a PPARγ-dependent switch from Lipogenesis to fat oxidation. Diabetes. 2015;64(7):2398–2408. doi:10.2337/db14-1213. 25695945

[115] Yamashita H, Fujisawa K, Ito E, Idei S, Kawaguchi N, Kimoto M, Hiemori M, Tsuji H. Improvement of obesity and glucose tolerance by acetate in type 2 diabetic otsuka long-Evans Tokushima fatty (OLETF) rats. Biosci Biotechnol Biochem. 2007;71(5):1236–1243. doi:10.1271/bbb.60668. 17485860

[116] Lin HV, Frassetto A, Kowalik Jr EJ Jr., Nawrocki AR, Lu MM, Kosinski JR, Hubert JA, Szeto D, Yao X, Forrest G, et al. Butyrate and propionate protect against diet-induced obesity and regulate gut hormones via free fatty acid receptor 3-independent mechanisms. PLoS One. 2012;7(4):e35240. doi:10.1371/journal.pone.0035240. 22506074 PMC3323649

[117] Frost G, Sleeth ML, Sahuri-Arisoylu M, Lizarbe B, Cerdan S, Brody L, Anastasovska J, Ghourab S, Hankir M, Zhang S, et al. The short-chain fatty acid acetate reduces appetite via a central homeostatic mechanism. Nat Commun. 2014;5(1):3611. doi:10.1038/ncomms4611. 24781306 PMC4015327

[118] Gao Z, Yin J, Zhang J, Ward RE, Martin RJ, Lefevre M, Cefalu WT, Ye J. Butyrate improves insulin sensitivity and increases energy expenditure in mice. Diabetes. 2009;58(7):1509–1517. doi:10.2337/db08-1637. 19366864 PMC2699871

[119] Sahuri-Arisoylu M, Brody LP, Parkinson JR, et al. Reprogramming of hepatic fat accumulation and ‘browning’ of adipose tissue by the short-chain fatty acid acetate. Int J Obes (Lond). 2016;40(6):955–963. doi:10.1038/ijo.2016.23. 26975441

[120] Steinert RE, Feinle-Bisset C, Asarian L, Horowitz M, Beglinger C, Ghrelin GN. CCK, GLP-1, and PYY(3-36): secretory controls and physiological roles in eating and glycemia in health, obesity, and after RYGB. Physiol Rev. 2017;97(1):411–463. doi:10.1152/physrev.00031.2014. 28003328 PMC6151490

[121] Guida C, Stephen SD, Watson M, Dempster N, Larraufie P, Marjot T, Cargill T, Rickers L, Pavlides M, Tomlinson J, et al. PYY plays a key role in the resolution of diabetes following bariatric surgery in humans. EBioMedicine. 2019;40:67–76. doi:10.1016/j.ebiom.2018.12.040. 30639417 PMC6413583

[122] Adam TC, Westerterp-Plantenga MS. Glucagon-like peptide-1 release and satiety after a nutrient challenge in normal-weight and obese subjects. Br J Nutr. 2005;93(6):845–851. doi:10.1079/bjn20041335. 16022753

[123] Batterham RL, Cohen MA, Ellis SM, Le Roux CW, Withers DJ, Frost GS, Ghatei MA, Bloom SR. Inhibition of food intake in obese subjects by peptide YY 3–36. N Engl J Med. 2003;349(10):941–948. doi:10.1056/NEJMoa030204. 12954742

[124] Zwirska-Korczala K, Konturek SJ, Sodowski M, Wylezol M, Kuka D, Sowa P, Adamczyk-Sowa M, Kukla M, Berdowska A, Rehfeld JF, et al. Basal and postprandial plasma levels of PYY, ghrelin, cholecystokinin, gastrin and insulin in women with moderate and morbid obesity and metabolic syndrome. J Physiol Pharmacol. 2007 [Accessed 2023 Aug 6]. 58(1):13–35. https://pubmed.ncbi.nlm.nih.gov/17443025/. 17443025

[125] Koliaki C, Liatis S, Dalamaga M, Kokkinos A. The implication of gut hormones in the regulation of energy homeostasis and their role in the pathophysiology of obesity. Curr Obes Rep. 2020;9(3):255–271. doi:10.1007/s13679-020-00396-9. 32647952

[126] Vaiserman A, Romanenko M, Piven L, Moseiko V, Lushchak O, Kryzhanovska N, Guryanov V, Koliada A. Differences in the gut Firmicutes to bacteroidetes ratio across age groups in healthy Ukrainian population. BMC Microbiol. 2020;20(1):221. doi:10.1186/s12866-020-01903-7. 32698765 PMC7374892

[127] Stojanov S, Berlec A, Strukelj B. The influence of probiotics on the firmicutes/bacteroidetes ratio in the treatment of obesity and inflammatory bowel disease. Microorganisms. 2020;8(11):1715. doi:10.3390/microorganisms8111715. 33139627 PMC7692443

[128] Ley RE, Backhed F, Turnbaugh P, Lozupone CA, Knight RD, Gordon JI. Obesity alters gut microbial ecology. Proc Natl Acad Sci USA. 2005;102(31):11070–11075. doi:10.1073/pnas.0504978102. 16033867 PMC1176910

[129] Sweeney TE, Morton JM. The human gut microbiome: a review of the effect of obesity and surgically induced weight loss. JAMA Surg. 2013;148(6):563–569. doi:10.1001/jamasurg.2013.5. 23571517 PMC4392891

[130] Koliada A, Syzenko G, Moseiko V, Budovska L, Puchkov K, Perederiy V, Gavalko Y, Dorofeyev A, Romanenko M, Tkach S, et al. Association between body mass index and firmicutes/bacteroidetes ratio in an adult Ukrainian population. BMC Microbiol. 2017;17(1):120. doi:10.1186/s12866-017-1027-1. 28532414 PMC5440985

[131] Crovesy L, Masterson D, Rosado EL. Profile of the gut microbiota of adults with obesity: a systematic review. Eur J Clin Nutr. 2020;74(9):1251–1262. doi:10.1038/s41430-020-0607-6. 32231226

[132] Krajmalnik-Brown R, Ilhan ZE, Kang DW, DiBaise JK. Effects of gut microbes on nutrient absorption and energy regulation. Nutr Clin Pract. 2012;27(2):201–214. doi:10.1177/0884533611436116. 22367888 PMC3601187

[133] Jumpertz R, Le DS, Turnbaugh PJ, Trinidad C, Bogardus C, Gordon JI, Krakoff J. Energy-balance studies reveal associations between gut microbes, caloric load, and nutrient absorption in humans. Am J Clin Nutr. 2011;94(1):58–65. doi:10.3945/ajcn.110.010132. 21543530 PMC3127503

[134] Zhang H, DiBaise JK, Zuccolo A, Kudrna D, Braidotti M, Yu Y, Parameswaran P, Crowell MD, Wing R, Rittmann BE, et al. Human gut microbiota in obesity and after gastric bypass. Proc Natl Acad Sci U S A. 2009;106(7):2365–2370. doi:10.1073/pnas.0812600106. 19164560 PMC2629490

[135] Duncan SH, Lobley GE, Holtrop G, Ince J, Johnstone AM, Louis P, Flint HJ. Human colonic microbiota associated with diet, obesity and weight loss. Int J Obes. 2008;32(11):1720–1724. doi:10.1038/ijo.2008.155. 18779823

[136] Hu HJ, Park SG, Jang HB, Choi M-G, Park K-H, Kang JH, Park SI, Lee H-J, Cho S-H, et al. Obesity alters the microbial community profile in Korean adolescents. PLoS One. 2015;10(7):e0134333. doi:10.1371/journal.pone.0134333. 26230509 PMC4521691

[137] Patil DP, Dhotre DP, Chavan SG, Sultan A, Jain DS, Lanjekar VB, Gangawani J, Shah PS, Todkar JS, Shah S, et al. Molecular analysis of gut microbiota in obesity among Indian individuals. J Biosci. 2012;37(4):647–657. doi:10.1007/s12038-012-9244-0. 22922190

[138] Abdallah Ismail N, Ragab SH, Abd Elbaky A, Shoeib AR, Alhosary Y, Fekry D. Frequency of firmicutes and bacteroidetes in gut microbiota in obese and normal weight Egyptian children and adults. Arch Med Sci. 2011;7(3):501–507. doi:10.5114/aoms.2011.23418. 22295035 PMC3258740

[139] Everard A, Belzer C, Geurts L, Ouwerkerk JP, Druart C, Bindels LB, Guiot Y, Derrien M, Muccioli GG, Delzenne NM, et al. Cross-talk between akkermansia muciniphila and intestinal epithelium controls diet-induced obesity. Proc Natl Acad Sci U S A. [2013 May 28]. 110(22):9066–9071. doi:10.1073/pnas.1219451110. 23671105 PMC3670398

[140] Karlsson CL, Onnerfalt J, Xu J, Molin G, Ahrne S, Thorngren-Jerneck K. The microbiota of the gut in preschool children with normal and excessive body weight. Obesity. 2012;20(11):2257–2261. doi:10.1038/oby.2012.110. 22546742

[141] Geerlings SY, Kostopoulos I, de Vos Wm, Belzer C, De Vos WM. Akkermansia muciniphila in the human gastrointestinal tract: when, where, and how? Microorganisms. 2018;6(3):75. doi:10.3390/microorganisms6030075. 30041463 PMC6163243

[142] Mishra SP, Wang B, Jain S, Ding J, Rejeski J, Furdui CM, Kitzman DW, Taraphder S, Brechot C, Kumar A, et al. A mechanism by which gut microbiota elevates permeability and inflammation in obese/diabetic mice and human gut. Gut. 2023;72(10):1848–1865. doi:10.1136/gutjnl-2022-327365. 36948576 PMC10512000

[143] Bervoets L, Van Hoorenbeeck K, Kortleven I, Van Noten C, Hens N, Vael C, Goossens H, Desager KN, Vankerckhoven V. Differences in gut microbiota composition between obese and lean children: a cross-sectional study. Gut Pathog. 2013;5(1):10. doi:10.1186/1757-4749-5-10. 23631345 PMC3658928

[144] Rizzatti G, Lopetuso LR, Gibiino G, Binda C, Gasbarrini A. Proteobacteria: a common factor in human diseases. Biomed Res Int. 2017;2017:9351507. doi:10.1155/2017/9351507. 29230419 PMC5688358

[145] Ratner AJ, Hippe KR, Aguilar JL, Bender MH, Nelson AL, Weiser JN. Epithelial cells are sensitive detectors of bacterial pore-forming toxins. J Biol Chem. 2006;281(18):12994–12998. doi:10.1074/jbc.M511431200. 16520379 PMC1586115

[146] Mendez-Salazar EO, Ortiz-Lopez MG, Granados-Silvestre MLA, Palacios-Gonzalez B, Menjivar M. Altered gut microbiota and compositional changes in firmicutes and proteobacteria in Mexican undernourished and obese children. Front Microbiol. 2018;9:2494. doi:10.3389/fmicb.2018.02494. 30386323 PMC6198253

[147] Lam YY, Ha CW, Hoffmann JM, Oscarsson J, Dinudom A, Mather TJ, Cook DI, Hunt NH, Caterson ID, Holmes AJ, et al. Effects of dietary fat profile on gut permeability and microbiota and their relationships with metabolic changes in mice. Obesity. 2015;23(7):1429–1439. doi:10.1002/oby.21122. 26053244

[148] Balamurugan R, George G, Kabeerdoss J, Hepsiba J, Chandragunasekaran AM, Ramakrishna BS. Quantitative differences in intestinal faecalibacterium prausnitzii in obese Indian children. Br J Nutr. 2010;103(3):335–338. doi:10.1017/S0007114509992182. 19849869

[149] Kulkarni P, Devkumar P, Chattopadhyay I. Could dysbiosis of inflammatory and anti-inflammatory gut bacteria have an implications in the development of type 2 diabetes? A pilot investigation. BMC Res Notes. 2021;14(1). doi:10.1186/s13104-021-05466-2. PMC7868023 33549142

[150] Palleja A, Kashani A, Allin KH, Nielsen T, Zhang C, Li Y, Brach T, Liang S, Feng Q, Jørgensen NB, et al. Roux-en-y gastric bypass surgery of morbidly obese patients induces swift and persistent changes of the individual gut microbiota. Genome Med. 2016;8(1):67. doi:10.1186/s13073-016-0312-1. 27306058 PMC4908688

[151] Farin W, Onate FP, Plassais J, Bonny C, Beglinger C, Woelnerhanssen B, Nocca D, Magoules F, Le Chatelier E, Pons N, et al. Impact of laparoscopic roux-en-y gastric bypass and sleeve gastrectomy on gut microbiota: a metagenomic comparative analysis. Surg Obes Relat Dis. 2020;16(7):852–862. doi:10.1016/j.soard.2020.03.014. 32360114

[152] Murphy R, Tsai P, Jullig M, Liu A, Plank L, Booth M. Differential changes in gut microbiota after gastric bypass and sleeve gastrectomy bariatric surgery vary according to diabetes remission. Obes Surg. 2017;27(4):917–925. doi:10.1007/s11695-016-2399-2. 27738970

[153] Al Assal K, Prifti E, Belda E, Sala P, Clément K, Dao M-C, Doré J, Levenez F, Taddei CR, Fonseca DC, et al. Gut microbiota profile of obese diabetic women submitted to roux-en-y gastric bypass and its association with food intake and postoperative diabetes remission. Nutrients. 2020;12(2):278. doi:10.3390/nu12020278. 31973130 PMC7071117

[154] Yu D, Shu XO, Howard EF, Long J, English WJ, Flynn CR. Fecal metagenomics and metabolomics reveal gut microbial changes after bariatric surgery. Surg Obes Relat Dis. 2020;16(11):1772–1782. doi:10.1016/j.soard.2020.06.032. 32747219 PMC9057387

[155] Kong LC, Tap J, Aron-Wisnewsky J, Pelloux V, Basdevant A, Bouillot J-L, Zucker J-D, Doré J, Clément K. Gut microbiota after gastric bypass in human obesity: increased richness and associations of bacterial genera with adipose tissue genes. Am J Clin Nutr. 2013;98(1):16–24. doi:10.3945/ajcn.113.058743. 23719559

[156] Kaniel O, Sherf-Dagan S, Szold A, Langer P, Khalfin B, Kessler Y, Raziel A, Sakran N, Motro Y, Goitein D, et al. The effects of one anastomosis gastric bypass surgery on the gastrointestinal tract. Nutrients. 2022;14(2):304. doi:10.3390/nu14020304. 35057486 PMC8778673

[157] Shen N, Caixas A, Ahlers M, Patel K, Gao Z, Dutia R, Blaser MJ, Clemente JC, Laferrère B. Longitudinal changes of microbiome composition and microbial metabolomics after surgical weight loss in individuals with obesity. Surg Obes Relat Dis. 2019;15(8):1367–1373. doi:10.1016/j.soard.2019.05.038. 31296445 PMC6722012

[158] Dao MC, Everard A, Aron-Wisnewsky J, Sokolovska N, Prifti E, Verger EO, Kayser BD, Levenez F, Chilloux J, Hoyles L, et al. Akkermansia muciniphila and improved metabolic health during a dietary intervention in obesity: relationship with gut microbiome richness and ecology. Gut. 2016;65(3):426–436. doi:10.1136/gutjnl-2014-308778. 26100928

[159] Furet JP, Kong LC, Tap J, Poitou C, Basdevant A, Bouillot J-L, Mariat D, Corthier G, Doré J, Henegar C, et al. Differential adaptation of human gut microbiota to bariatric surgery–induced weight loss. Diabetes. 2010;59(12):3049–3057. doi:10.2337/db10-0253. 20876719 PMC2992765

[160] Shahabi S, Mahmoudieh M, Keleidari B, Khodadadi H, Sheikhbahaei E, Shokrani Foroushani R. Gut firmicutes changes after laparoscopic one anastomosis gastric bypass surgery. Ann Bariatr Surg. 2019 [Accessed 2023 Aug 6]. 8(2):5. https://www.magiran.com/paper/2214654/gut-firmicutes-changes-after-laparoscopic-one-anastomosis-gastric-bypass-surgery?lang=en.

[161] Shi Q, Wang Q, Zhong H, Li D, Yu S, Yang H, Wang C, Yin Z. Roux-en-y gastric bypass improved insulin resistance via alteration of the human gut microbiome and alleviation of endotoxemia. Biomed Res Int. 2021;2021:1–14. doi:10.1155/2021/5554991. 34337024 PMC8294027

[162] Dao MC, Belda E, Prifti E, Everard A, Kayser BD, Bouillot J-L, Chevallier J-M, Pons N, Le Chatelier E, Ehrlich SD, et al. Akkermansia muciniphila abundance is lower in severe obesity, but its increased level after bariatric surgery is not associated with metabolic health improvement. Am J Physiol Endocrinol Metab. 2019;317(3):E446–E459. doi:10.1152/ajpendo.00140.2019. 31265324

[163] Ottman N, Geerlings SY, Aalvink S, de Vos Wm, Belzer C, de Vos WM. Action and function of akkermansia muciniphila in microbiome ecology, health and disease. Best Pract Res Clin Gastroenterol. 2017;31(6):637–642. doi:10.1016/j.bpg.2017.10.001. 29566906

[164] Paganelli FL, Luyer M, Hazelbag CM, Uh H-W, Rogers MRC, Adriaans D, Berbers R-M, Hendrickx APA, Viveen MC, Groot JA, et al. Roux-y gastric bypass and sleeve gastrectomy directly change gut microbiota composition independent of surgery type. Sci Rep. 2019;9(1):10979. doi:10.1038/s41598-019-47332-z. 31358818 PMC6662812

[165] Graessler J, Qin Y, Zhong H, Zhang J, Licinio J, Wong M-L, Xu A, Chavakis T, Bornstein AB, Ehrhart-Bornstein M, et al. Metagenomic sequencing of the human gut microbiome before and after bariatric surgery in obese patients with type 2 diabetes: correlation with inflammatory and metabolic parameters. Pharmacogenomics J. 2013;13(6):514–522. doi:10.1038/tpj.2012.43. 23032991

[166] Miquel S, Leclerc M, Martin R, Chain F, Lenoir M, Raguideau S, Hudault S, Bridonneau C, Northen T, Bowen B, et al. Identification of metabolic signatures linked to anti-inflammatory effects of faecalibacterium prausnitzii. mBio. 2015;6(2). doi:10.1128/mBio.00300-15. PMC4453580 25900655

[167] Li JV, Ashrafian H, Bueter M, Kinross J, Sands C, le Roux CW, Bloom SR, Darzi A, Athanasiou T, Marchesi JR, et al. Metabolic surgery profoundly influences gut microbial-host metabolic cross-talk. Gut. 2011;60(9):1214–1223. doi:10.1136/gut.2010.234708. 21572120 PMC3677150

[168] Juarez-Fernandez M, Roman-Saguillo S, Porras D, García-Mediavilla MV, Linares P, Ballesteros-Pomar MD, Urioste-Fondo A, Álvarez-Cuenllas B, González-Gallego J, Sánchez-Campos S, et al. Long-term effects of bariatric surgery on gut microbiota composition and faecal metabolome related to obesity remission. Nutrients. 2021;13(8):2519. doi:10.3390/nu13082519. 34444679 PMC8397959

[169] Pajecki D, de Oliveira LC, Sabino EC, de Souza-Basqueira M, Batista Dantas AC, Nunes GC, de Cleva R, Santo MA. Changes in the intestinal microbiota of superobese patients after bariatric surgery. Clinics. 2019;74:e1198. doi:10.6061/clinics/2019/e1198. 31664418 PMC6807688

[170] Jin Z, Chen K, Zhou Z, Peng W, Liu W. Roux-en-Y gastric bypass potentially improved intestinal permeability by regulating gut innate immunity in diet-induced obese mice. Sci Rep. 2021;11(1):14894. doi:10.1038/s41598-021-94094-8. 34290269 PMC8295358

[171] Wu J, Zhang PB, Ren ZQ, Zhou F, Hu H-H, Zhang H, Xue K-K, Xu P, Shao X-Q. Changes of serum lipopolysaccharide, inflammatory factors, and cecal microbiota in obese rats with type 2 diabetes induced by roux-en-Y gastric bypass. Nutrition. 2019;67-68:110565. doi:10.1016/j.nut.2019.110565. 31561205

[172] Lu C, Li Y, Li L, Kong Y, Shi T, Xiao H, Cao S, Zhu H, Li Z, Zhou Y, et al. Alterations of serum uric acid level and gut microbiota after roux-en-y gastric bypass and sleeve gastrectomy in a hyperuricemic rat model. Obes Surg. 2020;30(5):1799–1807. doi:10.1007/s11695-019-04328-y. 32124218 PMC7228899

[173] Guo Y, Liu CQ, Liu GP, Huang ZP, Zou DJ. Roux-en-y gastric bypass decreases endotoxemia and inflammatory stress in association with improvements in gut permeability in obese diabetic rats. J Diabetes. 2019;11(10):786–793. doi:10.1111/1753-0407.12906. 30714321

[174] Troseid M, Nestvold TK, Rudi K, Thoresen H, Nielsen EW, Lappegard KT. Plasma lipopolysaccharide is closely associated with glycemic control and abdominal obesity: evidence from bariatric surgery. Diabetes Care. 2013;36(11):3627–3632. doi:10.2337/dc13-0451. 23835694 PMC3816876

[175] Monte SV, Caruana JA, Ghanim H, Sia CL, Korzeniewski K, Schentag JJ, Dandona P. Reduction in endotoxemia, oxidative and inflammatory stress, and insulin resistance after roux-en-y gastric bypass surgery in patients with morbid obesity and type 2 diabetes mellitus. Surgery. 2012;151(4):587–593. doi:10.1016/j.surg.2011.09.038. 22088821

[176] Turner L, Santosa S. Putting atm to bed: how adipose tissue macrophages are affected by bariatric surgery, exercise, and dietary fatty acids. Adv Nutr. 2021;12(5):1893–1910. doi:10.1093/advances/nmab011. 33979430 PMC8483961

[177] Harma MA, Adeshara K, Istomin N, Lehto M, Blaut M, Savolainen MJ, Hörkkö S, Groop P-H, Koivukangas V, Hukkanen J, et al. Gastrointestinal manifestations after roux-en-Y gastric bypass surgery in individuals with and without type 2 diabetes. Surg Obes Relat Dis. 2021;17(3):585–594. doi:10.1016/j.soard.2020.10.021. 33246847

[178] Russell WR, Gratz SW, Duncan SH, Holtrop G, Ince J, Scobbie L, Duncan G, Johnstone AM, Lobley GE, Wallace RJ, et al. High-protein, reduced-carbohydrate weight-loss diets promote metabolite profiles likely to be detrimental to colonic health. Am J Clin Nutr. 2011;93(5):1062–1072. doi:10.3945/ajcn.110.002188. 21389180

[179] Duncan SH, Belenguer A, Holtrop G, Johnstone AM, Flint HJ, Lobley GE. Reduced dietary intake of carbohydrates by obese subjects results in decreased concentrations of butyrate and butyrate-producing bacteria in feces. Appl Environ Microbiol. 2007;73(4):1073–1078. doi:10.1128/AEM.02340-06. 17189447 PMC1828662

[180] Mukorako P, Lemoine N, Biertho L, Lebel S, Roy M-C, Plamondon J, Tchernof A, Varin TV, Anhê FF, St-Pierre DH, et al. Consistent gut bacterial and short-chain fatty acid signatures in hypoabsorptive bariatric surgeries correlate with metabolic benefits in rats. Int J Obes (Lond). 2022;46(2):297–306. doi:10.1038/s41366-021-00973-5. 34686781

[181] Roushdy A, Abdel-Razik MA, Emile SH, Farid M, Elbanna HG, Khafagy W, Elshobaky A. Fasting ghrelin and postprandial glp-1 levels in patients with morbid obesity and medical comorbidities after sleeve gastrectomy and one-anastomosis gastric bypass: a randomized clinical trial. Surg Laparosc Endosc Percutan Tech. 2020;31(1):28–35. doi:10.1097/SLE.0000000000000844. 32810030

[182] Marciniak C, Chavez-Talavera O, Caiazzo R, Hubert T, Zubiaga L, Baud G, Quenon A, Descat A, Vallez E, Goossens JF, et al. Characterization of one anastomosis gastric bypass and impact of biliary and common limbs on bile acid and postprandial glucose metabolism in a minipig model. Am J Physiol Endocrinol Metab. 2021;320(4):E772–E783. doi:10.1152/ajpendo.00356.2020. 33491532 PMC8906817

[183] Bednarz K, Kowalczyk K, Cwynar M, Czapla D, Czarkowski W, Kmita D, Nowak A, Madej P. The role of glp-1 receptor agonists in insulin resistance with concomitant obesity treatment in polycystic ovary syndrome. Int J Mol Sci. 2022;23(8):4334. doi:10.3390/ijms23084334. 35457152 PMC9029608

[184] Witjaksono F, Lukito W, Wijaya A, Annisa NG, Jutamulia J, Nurwidya F, Simadibrata M. The effect of breakfast with different macronutrient composition on PYY, ghrelin, and ad libitum intake 4 h after breakfast in Indonesian obese women. BMC Res Notes. 2018;11(1):787. doi:10.1186/s13104-018-3895-3. 30390699 PMC6215622

[185] van Rijswijk A, van Olst N, Meijnikman AS, van Rijswijk A, van Olst N, Acherman YIZ, Bruin SC, van de Laar AW, van Olden CC, Aydin O, et al. The effects of laparoscopic roux-en-Y gastric bypass and one-anastomosis gastric bypass on glycemic control and remission of type 2 diabetes mellitus: study protocol for a multi-center randomized controlled trial (the diabar-trial). Trials. 2022;23(1):900. doi:10.1186/s13063-022-06762-3. 36273149 PMC9588204

